# Seeking optimal non-pharmacological interventions for sarcopenia: a systematic review and network meta-analysis

**DOI:** 10.1007/s40520-024-02920-6

**Published:** 2025-01-15

**Authors:** Zhenyue Fu, Yajiao Wang, Lu Zhao, Yumeng Li, Qingqiao Song

**Affiliations:** 1 Department of General Internal Medicine, Guang’anmen Hospital, China Academy of Chinese Medical Sciences, Beijing, China; 2 Beijing University of Chinese Medicine, Beijing, China

**Keywords:** Sarcopenia, Non-pharmacological interventions, Systematic review, Network meta-analysis

## Abstract

**Background:**

With the acceleration of aging, sarcopenia has become a reality of concern today. This study aimed to evaluate the efficacy of various non-pharmacologic interventions and find the optimal interventions for sarcopenia.

**Methods:**

PubMed, Medline OVID, EMBASE, Scopus, and Cochrane were searched from 1 January 2000 to 25 October 2023, with language restrictions to English. We analyzed the data through the Bayesian network meta-analysis.

**Results:**

Based on the inclusion and exclusion criteria defined by the PICOS principles, we identified 47 eligible clinical trials engaging 4889 individuals (including treatment group = 2835, control group = 2054). The results showed that resistance exercise (low-moderate load) significantly increased muscle mass (skeletal muscle mass and lean body mass) and that exercise plus nutrition improved physical activity indices (handgrip strength, gait speed, TUG test, chair standing).

**Conclusion:**

Resistance exercise (low-moderate load), exercise plus nutrition, and nutritional supplementation (fatty acids, etc.) may be protective against sarcopenia.

*Systematic Review Registration* https://www.crd.york.ac.uk/prospero/display_record.php?RecordID=474799, ID: CRD4202347479.

**Supplementary Information:**

The online version contains supplementary material available at 10.1007/s40520-024-02920-6.

## Introduction

With the increasing tendency of overall aging, gerontology has seen rapid development in recent decades. The term “Sarcopenia” was born in 1989, and consists of the Greek words sarx (flesh) and penia (loss) [1]. As a progressive silent disease, sarcopenia refers to a spiral decline in skeletal muscle mass and strength with age. Recent epidemiologic surveys have shown a prevalence of sarcopenia in the general population of approximately 5–22% based on EWGSOP/ EWGSOP2 diagnostic criteria and 8–15% based on AWGS diagnostic criteria [2, 3], all-cause mortality can increase by 60% to 260% [4, 5]. Meanwhile, muscle loss is accompanied by a decline in physical activity, which amplifies the risk of a wide range of age-related cardiometabolic diseases, cancers and neurodegenerative disorders [6]. However, during clinical practice, the difficulty in recognizing sarcopenia, inconsistent diagnostic criteria, and lack of care directly contribute to its severe neglect and under-treatment.

Muscle loss is an inevitable part of aging, and numerous scientific researchers and clinicians are closely monitoring how to delay its onset and decelerate its progression. Unfortunately, there is currently no FDA-approved medication for the treatment of sarcopenia. The 2018 International Conference on Frailty and Sarcopenia Research (ICFSR) [7] issued a guideline stating that progressive resistance-based training prescription and protein supplementation/protein-rich diets are recommended intervention protocols with a grade of evidence, and the PROT-AGE working group identified a protein intake of 1.0–1.2 g/kg body weight to maintain muscle mass and physical function [8], corroborating the importance of non-pharmacological interventions for sarcopenia management. Existing meta-analysis evidence summarizes the effects of different exercise modes (resistance/aerobic/combined), exercise doses, and nutritional supplements (protein, vitamins, unsaturated fatty acids) on the management of sarcopenia [9–12] and draws different conclusions. A network meta-analysis by Shen et al. demonstrated that the combination of resistance exercise with aerobic and balance training can significantly improve the quality of life and physical function in older adults with sarcopenia [13]. Whereas, Kamińska et al. found that whey protein did not modulate the parameters associated with sarcopenia [14]. However, the evidence for combined non-pharmacological interventions has not yet been summarized.

Based on the wide variety of nonpharmacologic therapies, it is difficult for traditional meta-analysis to synthesize evidence to determine the effectiveness and ranking of different nonpharmacologic therapies. Based on the principles of Bayesian statistics, Bayesian network meta-analysis estimates parameters by analyzing more than two interventions at the same time, combining prior knowledge and current data to provide complete probability distributions about treatment effects [15]. Therefore, this systematic evaluation and network meta-analysis aimed to (1) assess the efficacy of different forms of non-pharmacological interventions to improve anthropometric and physical performance indicators in patients with sarcopenia, and (2) construct an effective hierarchy of non-pharmacological interventions to find the optimal solution and paradigm of non-pharmacological interventions in sarcopenia, to give health advice on the lifestyles and dietary habits of older adults, and to provide more complementary therapies for patients with sarcopenia in clinical settings.

## Materials and methods

This network meta-analysis was conducted and reported in strict compliance with the Cochrane Handbook and the 27 entries listed in PRISMA-NMA (Appendix S1) [16]. We had registered it in the PROSPERO (International Prospective Registry of Systematic Reviews, ID: CRD42023474799) Literature retrieval, inclusion, data extraction, and quality assessment were performed simultaneously and independently by two researchers. In case of disagreement, consensus was reached through discussion or consultation with a third researcher.

### Information sources and retrieval strategy

Data traceability was restricted from 1 January 2000 to 25 October 2023, and the language was restricted in English. An expanded and systematic literature search was performed in 5 electronic databases: PubMed, Medline OVID, EMBASE, Scopus, and Cochrane (Appendix S2). Supplementary searches were conducted on the National Institutes of Health (http://clinicaltrials.gov/), and the WHO International Clinical Trials Registry Platform (ICTRP, http://apps.who.int/trialsearch/) for ongoing clinical studies. In addition, reference lists of included studies and published systematic reviews and meta-analyses were hand-searched to improve coverage. This systematic review and network meta-analysis culminated in the inclusion of completed and publicly published clinical trials.

### Eligibility criteria

The inclusion criteria were strictly based on the PICOS principles and included the following entries:

1. Participants: individuals with sarcopenia identified by diagnostic criteria, including but not limited to EWGSOP1/2 [17], AWGS [18], the Foundation for the National Institutes of Health Biomarkers Consortium Sarcopenia Project (FNIH) [19].
2. Intervention: non-pharmacological treatment, including exercise, nutrition, comprehensive prescription, and other prescription. (1) Exercise prescription: according to the FIIT exercise prescription principles [20], it was categorized into aerobic exercise (AE, which is aimed at strengthening cardiorespiratory fitness and burning fat and energy, and includes walking, running, or biking), resistance exercise (RE, which is aimed at stimulating the skeletal musculature system and improving muscular strength and muscular endurance), combination exercise (CE, a combination of at least two exercises, such as AE, RE, and balance training), and whole body vibration exercise (designed to deliver impulsive stimuli to the limbs through a vibration platform to increase muscle activation and excitability). Resistance exercise is categorized according to its intensity (average maximum load) > 75% of the 1RM atmosphere high load and low-moderate load. (2) Nutrition prescription: according to the type of nutrient supplementation is categorized into proteins/amino acids, vitamins, and supplement mixes. (3) Comprehensive prescription: simultaneous exercise intervention and nutritional intervention. (4) Other prescription: e.g., acupuncture, tai chi, etc.
3. Control: The control group did not receive interventions or use measures such as a placebo, standard treatment, and health education that had no evidence of influencing outcome indicators.
4. Outcome: Several primary and secondary outcomes related to the symptoms of sarcopenia were identified according to the recently published EWGSOP2 consensus. Primary outcomes: muscle mass (skeletal muscle mass and lean body mass) assessed by magnetic resonance imaging (MRI), dual-energy X-ray absorptiometry (DEXA), bioelectrical impedance analysis (BIA), and computed tomography (CT) imaging. Secondary outcomes: physical measures (body weight, BMI), physical activity [handgrip strength, gait speed, Timed Up and Go test (TUG), chair standing test].
5. Study design: Randomized controlled trials, including parallel and crossover designs.

The exclusion criteria were:

1. Participants with tumors, severe cardiac insufficiency (NYHA > II), chronic kidney disease, abnormal liver function, chronic obstructive pulmonary disease, gastrointestinal disorders, or who were pregnant, breastfeeding, or smoked cigarettes or drank alcohol;
2. Studies with inaccessible outcome indicators;
3. Reviews, commentaries, conference abstracts, case reports.

### Data extraction

The samples and analysis results of intention-to-treat (ITT) were preferentially used. Construct a standardized data extraction form and enter the following information: (1) Basic information: first author's name, nationality, year of publication, trial registration number, diagnostic criteria, and source of subjects (hospital/community/nursing home); (2) Baseline information: sample size of the intervention group versus the control group, male-to-female ratio, average age, BMI health status, and baseline disease; (3) Study content information: non-pharmacological interventions or placebo content, frequency, duration of intervention; (4) Outcome information: primary and secondary outcomes. (5) Trial process: study design, randomization method, implementation of allocation concealment, blinding format.

### Risk of bias

The included studies were evaluated and analyzed according to the Cochrane Risk of Bias V.2.0. tool [21, 22]. Evaluation entries included: the generation of randomized sequences, allocation concealment, blinding, selection bias, incomplete outcome information, selective reporting of outcomes, and other sources of bias. The risk of bias for each entry was evaluated as “low risk”, “high risk”, and “unclear risk”. The confidence in network meta-analysis primary outcome was assessed using the confidence in network meta-analysis (CINeMA) framework (https://cinema.ispm.unibe.ch/) constructed based on GRADE grading [23].

### Network meta-analysis

A network meta-analysis model based on a Bayesian framework was constructed using the “Gemtc” package in R software (version 4.2.0; R Foundation, Vienna, Austria) for data processing and analysis [24]. The data were all continuous variables, and the units of each outcome were consistent, so the effect sizes were presented as mean difference (MD) and 95% CI (confidence interval). If the outcome data were presented as median/interquartile range (IQR)/minimum and maximum in the original literature, they were converted to Mean and SD using the formulae [25, 26]. Network plots were constructed to visualize the direct and indirect relationships between different interventions, with node size and line thickness proportional to the number of participants and studies. The effectiveness of interventions for each outcome was ranked using the surface under the cumulative ranking curve (SUCRA) and ranking probability plots, with a larger surface under the SUCRA curve (0%–100%) indicating a higher likelihood of the treatment being the optimal intervention [27].

Meta-regression was performed on the primary outcome indicators. Generated funnel plots were assessed in Stata 17.0 software to assess publication bias for the primary outcome by looking at the symmetry of the funnel plots [10, 28].

## Results

### Research screening and description

**Fig. 1:**
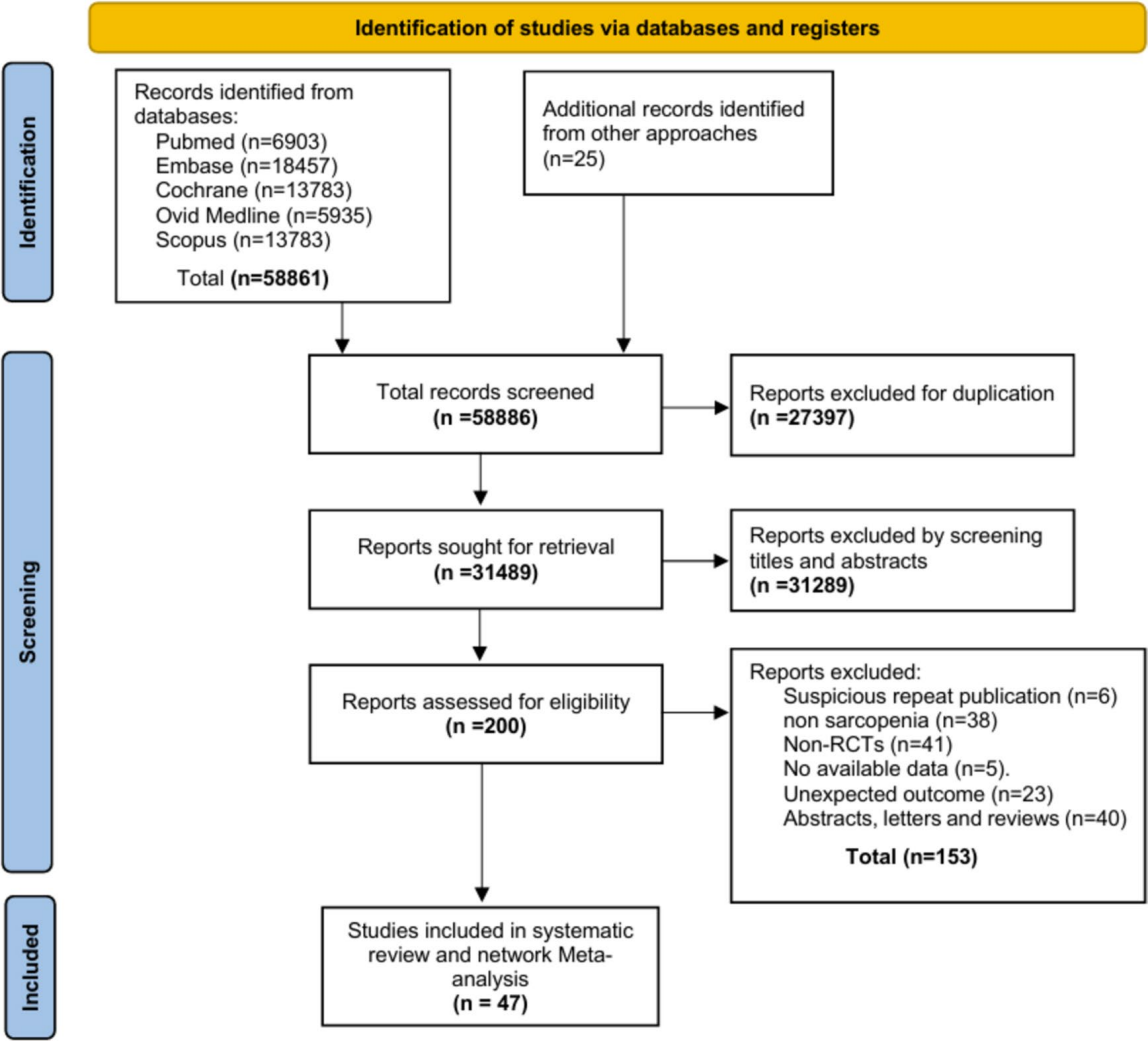
Flow chart of article screening

An expanded search strategy was used to avoid omissions, and a total of 58,886 articles were retrieved. After software and manual de-weighting, 31,489 articles remained, and 31,289 irrelevant articles were eliminated by reading titles and abstracts. The remaining 200 articles were read in full text and 153 articles did not fulfill the criteria. Data extraction, systematic review and network meta-analysis was performed on the remaining 47 articles (Fig. 1).

### Basic characterization

A total of 4889 participants were included in this systematic evaluation and network meta-analysis. All studies were conducted in a parallel randomized controlled trial design, with most studies conducted in Asia (n = 30) and Europe (n = 13), and a few in South America (n = 3) and Africa (n = 1). 35 studies received funding from industry, government, and institutions. 26 studies were registered in clinical trial registry sites, and 15 reported outcomes using an intention-to-treat analysis (ITT). Diagnostic criteria used in the included studies varied and included EWGSOP1/2 (n = 7), AWGS (n = 12), FNIH (n = 2), and customized sarcopenia criteria (n = 25). The age range of the included patients was 60–80 (n = 37), mostly recruited from community, hospital, and institution (Appendix S3).

### Non-pharmacological therapeutic interventions

Based on the FIIT exercise prescription and the rest of the non-pharmacological therapy categorization principles, we have attributed a total of 11 categories of interventions. Five types of exercise prescriptions [resistance exercise (low-moderate load), resistance exercise (high load), aerobic exercise, combined exercise (low-moderate load), whole-body vibration exercise], four types of nutrition prescription (protein, other nutrition, protein plus vitamins, mixed nutrition), one type of comprehensive prescription (exercise plus nutrition), and one type of other prescription (acupuncture) (Appendix S3).

### Quality assessment

According to the Cochrane handbook, the major threat to the quality grade was the lack of detailed descriptions of blinding [including participants, investigators (high risk of 59.6%), and outcome evaluation (high risk of 53.2%)]. Second, the generation of randomized sequences (unclear risk 42.6%) and allocation concealment (unclear risk 55.3%) were poorly described. Meanwhile, other risks of bias were unclear due to poor description of sample size calculation and inappropriate description of withdrawal. In conclusion, 20 studies were at high risk, 8 at low risk, and the rest at moderate risk.

The CINeMA assessment showed that for the outcome of skeletal muscle mass, four comparisons were at high risk, one comparison was at moderate risk, and two comparisons each were at low risk and very low risk. For the outcome of lean body mass, three comparisons were at high risk, four comparisons were at moderate risk, and one comparison was at low risk (Appendix S4).

### Network meta-analysis

#### Primary outcomes

**Fig. 2:**
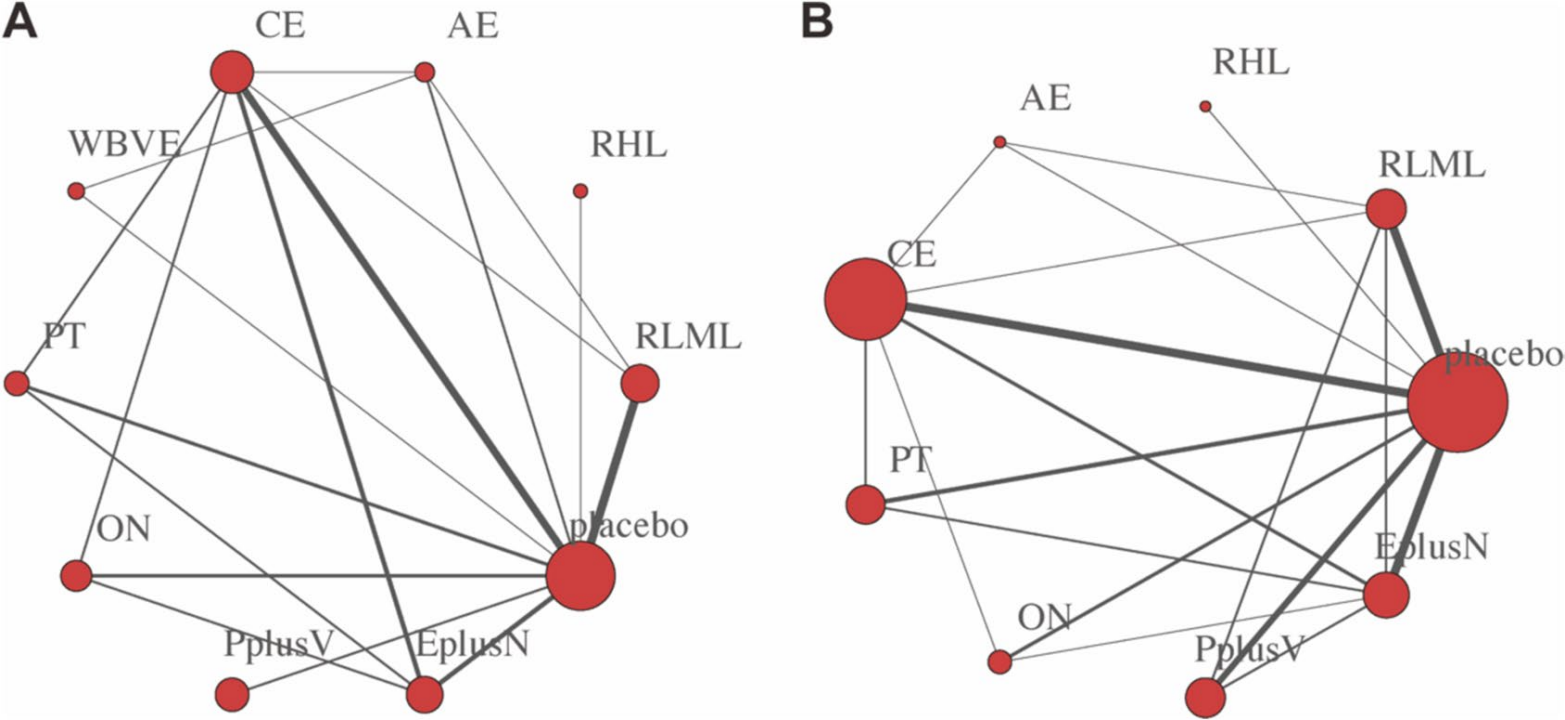
Network plot for the outcomes of **A** skeletal muscle mass and **B** lean body mass. Each node represents an intervention and node size is measured by the number of participants. The thickness of each edge (the line connecting the nodes) represents a direct comparison between interventions, and the thickness of the edge is measured by the number of participating studies. *RLML* resistance exercise (low-moderate load), *RHL* resistance (high load), *AE* aerobic exercise, *CE* combined exercise, *WBVE* whole-body vibration exercise, *PT* protein, *ON* other nutrition, *PplusV* protein plus vitamin, *EplusN* exercise plus nutrition

18 studies involved skeletal muscle mass (12 two-arm studies, 1 three-arm study, and 5 four-arm studies) and used 10 interventions. The 10 interventions included resistance exercise (low-moderate load), resistance (high load), aerobic exercise, combined exercise, whole-body vibration exercise, protein, other nutrition, protein plus vitamin, exercise plus nutrition. Nine studies addressed lean body mass (including 17 two-arm studies, 1 three-arm study, and 6 four-arm studies) and used nine interventions. The nine interventions included resistance exercise (low-moderate load), resistance (high load), aerobic exercise, combined exercise, protein, other nutrition, protein plus vitamin, exercise plus nutrition (Fig. 2).

**Fig. 3:**
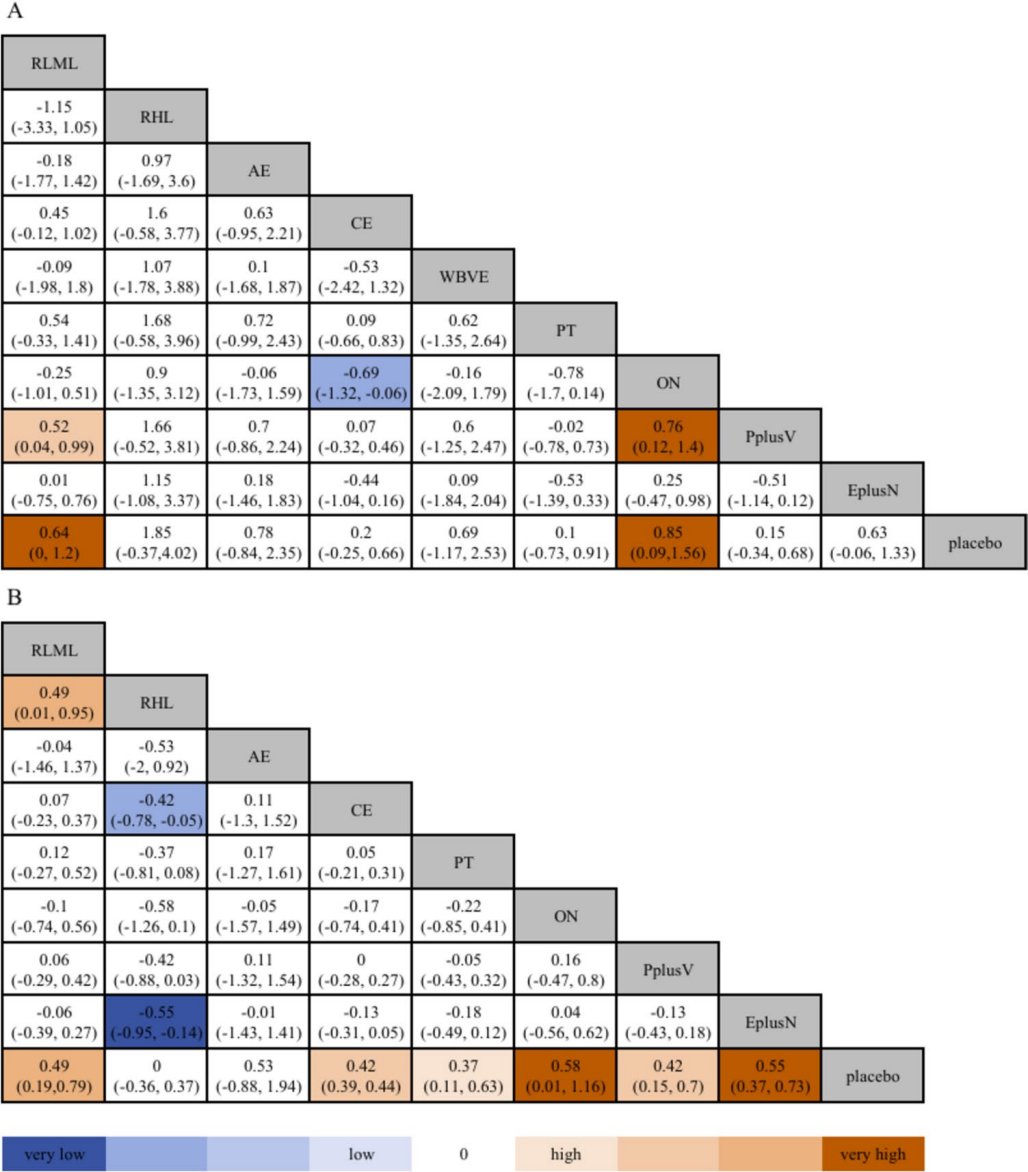
League Table of the primary outcomes. **A** League Table of skeletal muscle mass; **B** League Table of lean body mass. *RLML* resistance exercise (low-moderate load), *RHL* resistance (high load), *AE* aerobic exercise, *CE* combined exercise, *WBVE* whole-body vibration exercise, *PT* protein, *ON* other nutrition, *PplusV* protein plus vitamin, *EplusN* exercise plus nutrition

Compared with placebo, resistance exercise (low-moderate load) with other nutrition supplementation increased skeletal muscle mass [MD 0.64, 95% CI (0, 1.2); MD 0.85, 95% CI (0.09, 1.56)]. Resistance exercise (low-moderate load), combined exercise, protein, other nutrition, protein plus vitamin, and exercise plus nutrition [MD 0.49, 95% CI (0.19, 0.79); MD 0.42, 95% CI (0.39, 0.44); MD 0.37, 95% CI (0.11, 0.63); MD 0.58, 95% CI (0.01, 1.16); MD 0.42, 95% CI (0.15, 0.7); MD 0.55, 95% CI (0.37, 0.73)] increased lean body mass (Fig. 3).

SUCRA showed that the top three non-pharmacological interventions with the optimal efficacy in increasing skeletal muscle mass may be resistance (high load) (86% likelihood), aerobic exercise (62.7%), and resistance exercise (low-moderate load) (62.6%). The top three non-pharmacological interventions in increasing lean body mass are probably exercise plus nutrition (86% likelihood), other nutrition (62.7%), and resistance exercise (low-moderate load) (62.6%) (Appendix S6).

**Fig. 4:**
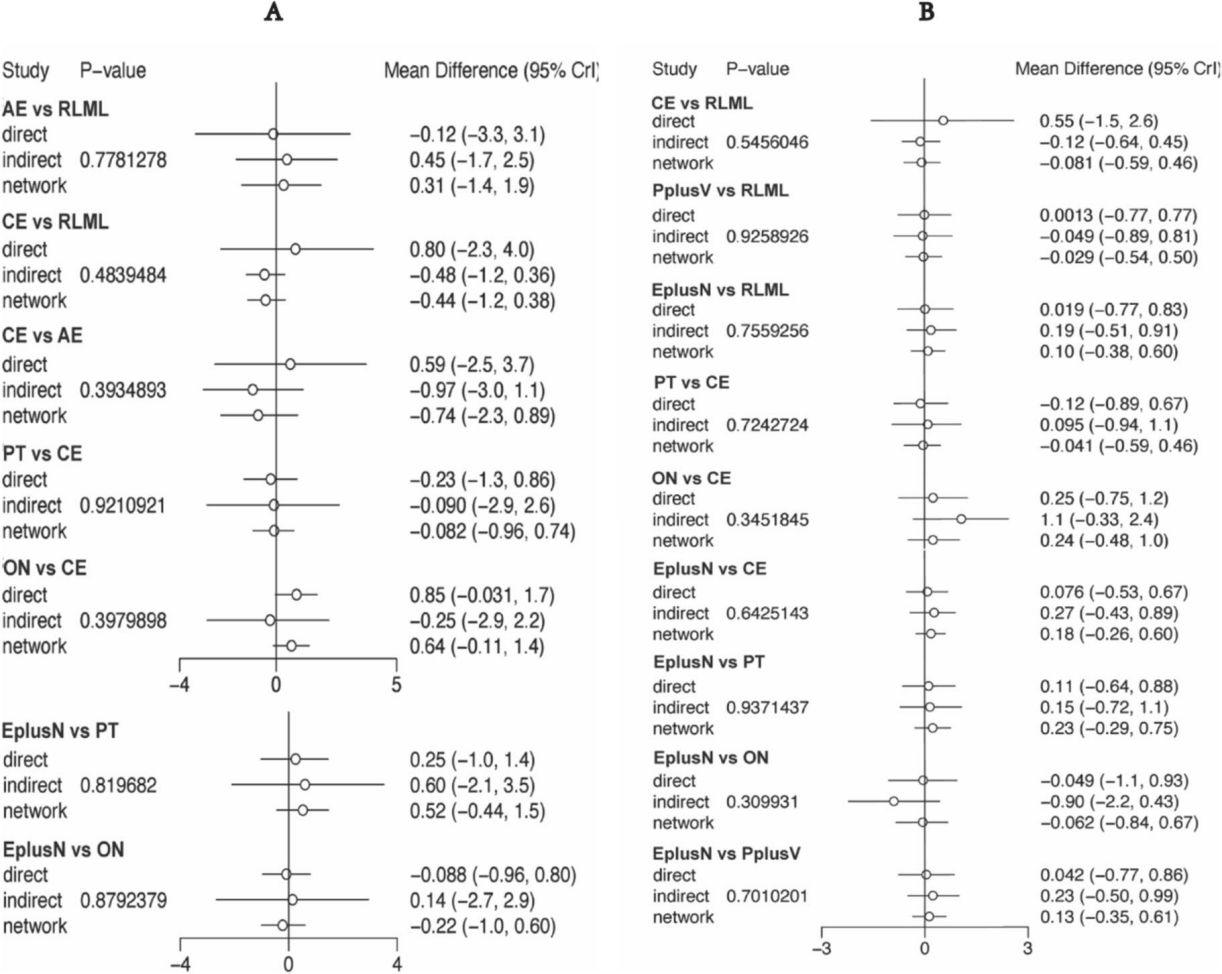
Forest plot and node-splitting model assessing incoherence between direct and indirect comparisons. **A** Skeletal muscle mass. **B** Lean body mass

The forest plots show no statistical differences between the direct and indirect comparisons of the different interventions on the main outcome indicators (Fig. 4).

#### Secondary outcomes

##### Physical activity indicators

**Fig. 5:**
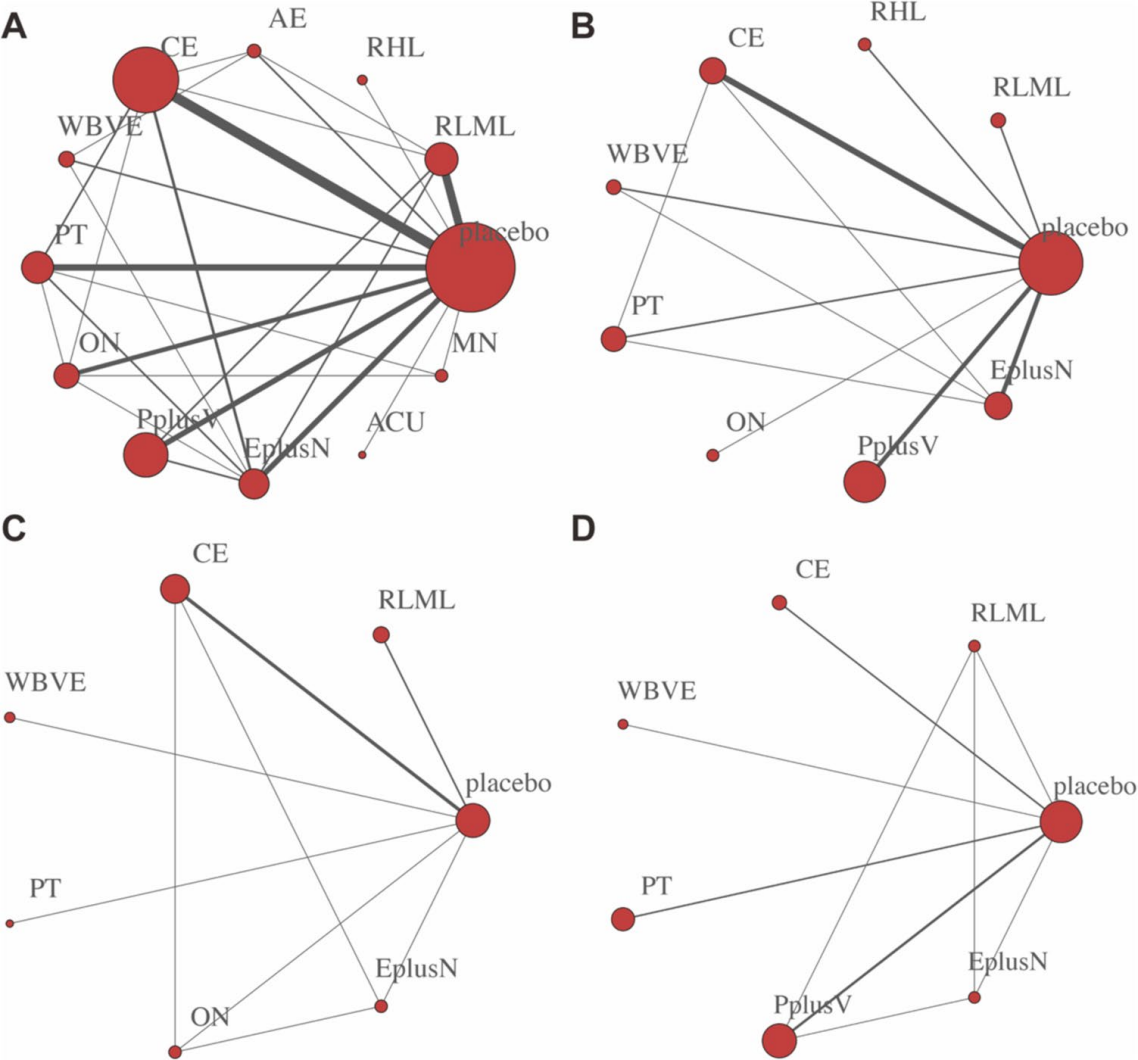
Network plot for the outcomes of **A** handgrip strength, **B** gait speed, **C** TUG test, and **D** chair standing

Thirty-five studies involved d handgrip strength (including 26 2-arm studies, 2 3-arm studies, and 7 4-arm studies) and used 12 interventions. Twenty-three studies involved gait speed (including 21 2-arm studies, 1 3-arm study, and 1 4-arm study) and used nine interventions. Eight studies involved the TUG test (seven 2-arm studies and one 4-arm study) and used seven interventions. Eight studies involved chair standing test(including seven 2-arm studies and one 4-arm study) and used seven interventions (Fig. 5).

SUCRA showed that the top three non-pharmacological interventions for increasing handgrip strength were probably resistance(low-moderate load) (78.6%) resistance exercise (high load) (74.6%), and other nutrition (66.7%); the three nonpharmacologic therapies with the best efficacy in increasing gait speed were probably resistance(low-moderate load) (96.7%), combined exercise (85.1%), and whole-body vibration exercise (73.4%); the top three non-pharmacological treatments for decreasing TUG test completion time were probably whole-body vibration exercise (86.5%), combined exercise (77%), and resistance exercise(low-moderate load) (63.5%); the top three nonpharmacologic therapies for decreasing chair standing completion time were probably other nutrition (86.6%), exercise plus nutrition (68.1%), and aerobic exercise (46.6%) (Appendix S6).

**Fig. 6:**
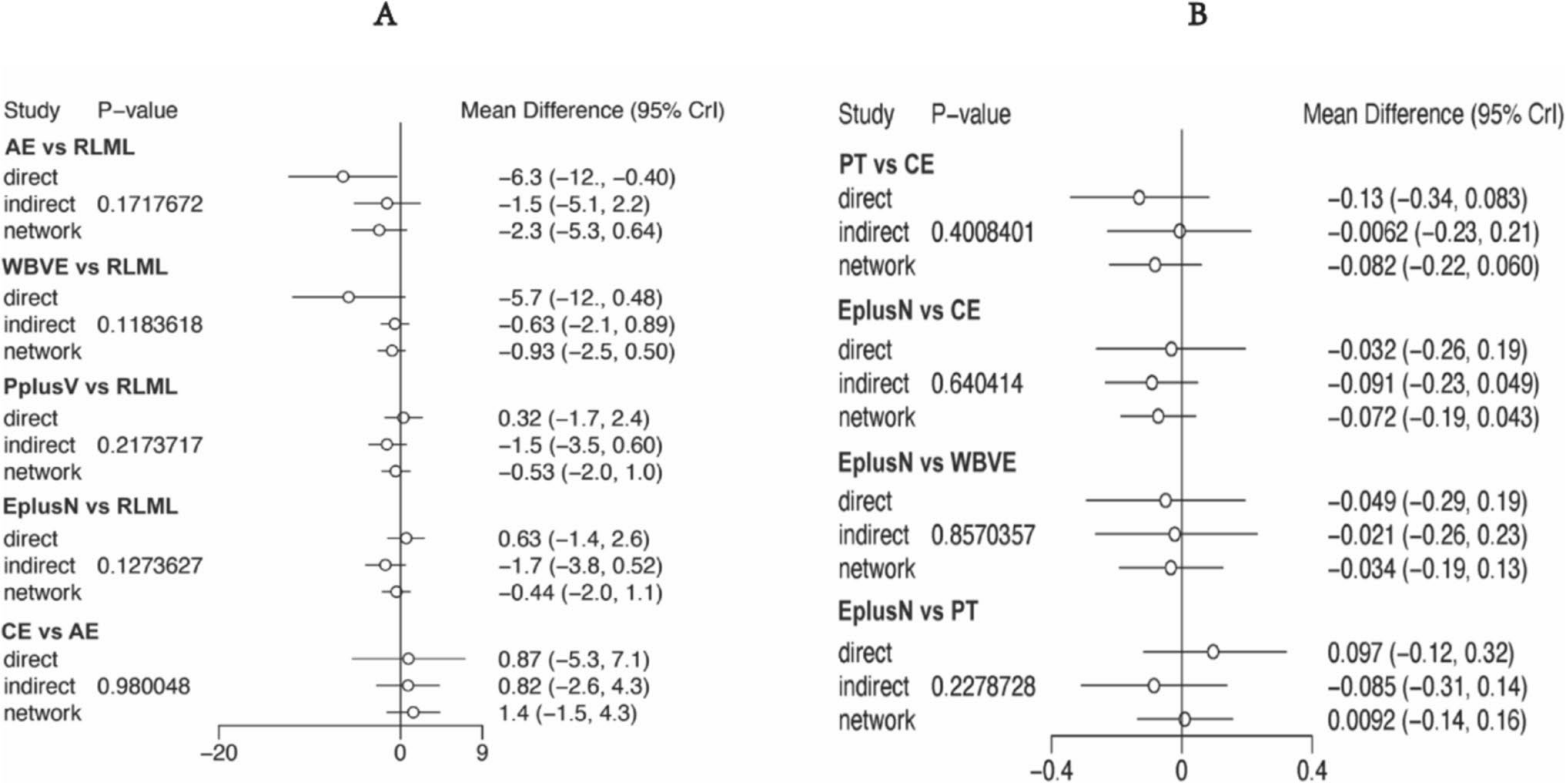
Forest plot and node-splitting model assessing incoherence between direct and indirect comparisons. **A** Handgrip strength. **B** Gait speed

The forest plots show no statistical differences between the direct and indirect comparisons of the different interventions on the physical activity indicators (Fig. 6).

##### Physical measures indicators

**Fig. 7:**
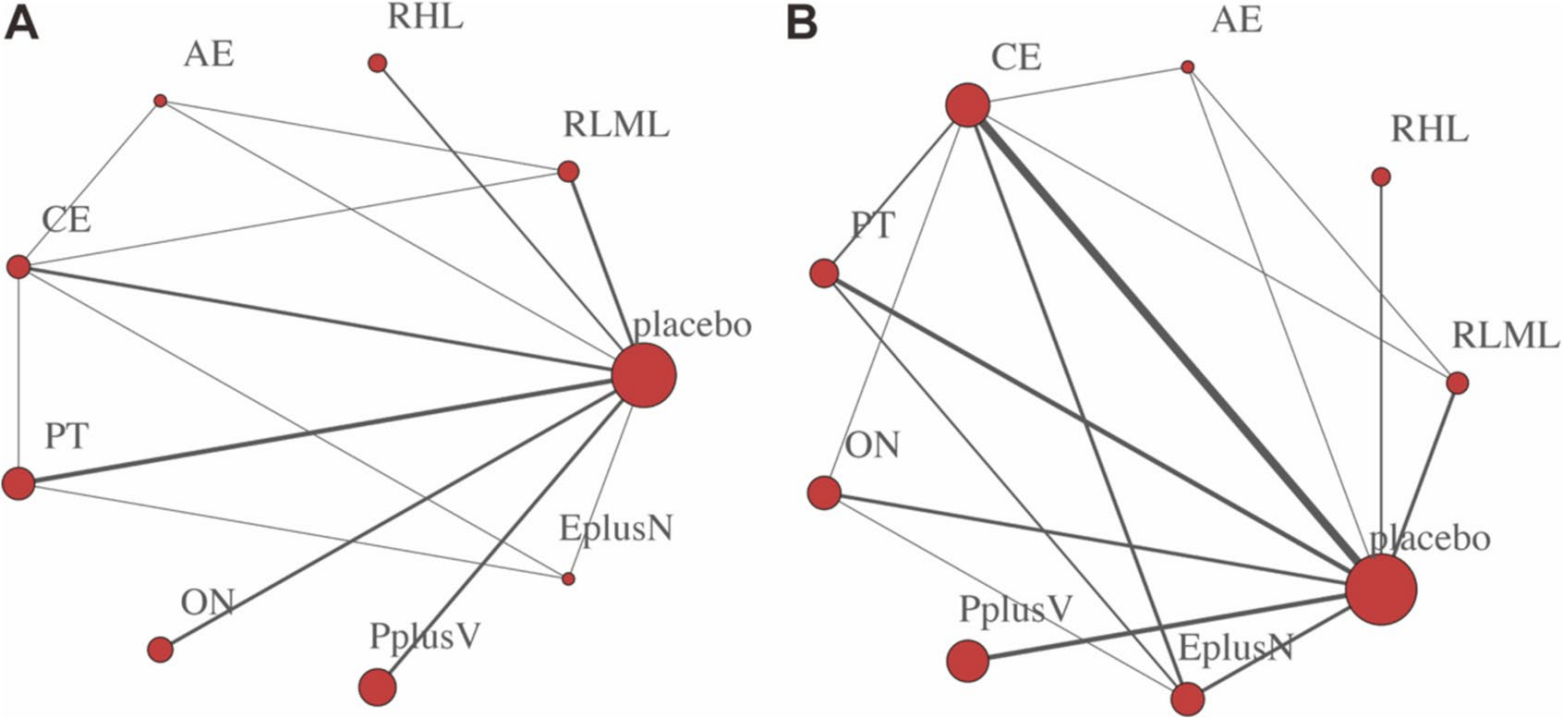
Network plot for the outcomes of **A** body weight and **B** BMI

Physical measures were measured by body weight and body mass index. According to the network plot, sixteen studies involved body weight (including twelve 2-arm studies and four 4-arm studies) and used nine interventions. Nineteen studies involved BMI (fifteen 2-arm studies and four 4-arm studies) and used nine interventions (Fig. 7).

**Fig. 8:**
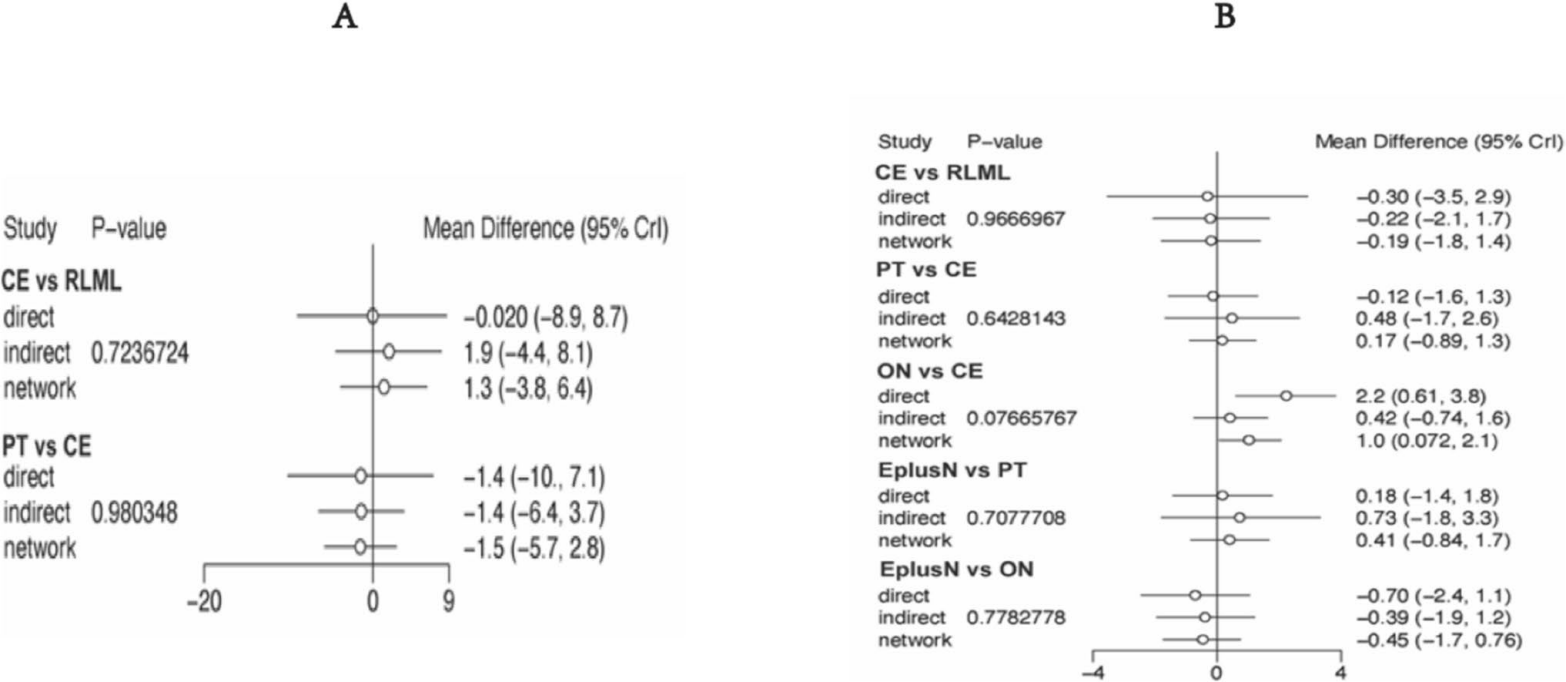
Forest plot and node-splitting model assessing incoherence between direct and indirect comparisons. **A** body weight. **B** BMI

Among the 19 studies that involved BMI, there was a difference between direct and indirect comparisons for other nutrition vs combined exercise [MD 2.2, 95% CI (0.61, 3.8)], and the remaining comparisons were not statistically different (Fig. 8).

#### Inconsistency and heterogeneity

According to the global I^2^, the heterogeneity of each outcome indicator in this study was relatively low (I^2^ < 10%). We applied the NMA model and UME model to each outcome index and found that the DIC fluctuated more among the six outcome indexes (lean body mass, handgrip strength, gait speed, body weight, body mass index, and SPPB) fluctuated greatly(difference > 10), suggesting the possible existence of global inconsistency (Appendix S5). The node-splitting method for each outcome did not detect the presence of local inconsistency between direct and indirect comparisons (*p* > 0.5).

#### Meta-regression and sensitivity analysis

Performing sensitivity analyses for the primary outcomes, we observed the symmetrical presence of scatter on both sides of the funnel plot. To further detect the presence of heterogeneity, we performed meta-regressions on covariates for the following potential sources of heterogeneity: mean participant age, total number of participants, baseline BMI, diagnostic criteria classification, and treatment duration. The presence of significant heterogeneity was not detected (Appendix S7).

## Discussion

### Overview of results

It is a Bayesian network meta-analysis integrating direct and indirect evidence from 11 non-pharmacological interventions to assess their effects on outcomes such as muscle mass, physical activity, quality of life, and physical measures in patients with sarcopenia. Based on the combined evidence, we found that resistance exercise (low-moderate load) significantly improved muscle mass (skeletal muscle mass and lean body mass) in various instrumental measurements (MRI, DEXA, BIA, CT) and improved some of the physical activity indices (handgrip strength, gait speed, TUG test). Exercise plus nutrition increased lean body mass and improved all physical activity indicators. Nutritional supplements such as fatty acids can increase muscle mass and improve quality of life scores. The above three are the top three ranked interventions that may be the optimal intervention in management of sarcopenia. However, the robustness of the findings is threatened by the small study sample size, high risk of quality assessment, and global inconsistency. Therefore, further studies are needed to reinforce our current conclusions.

### Overview of potential mechanisms

Older adults with many comorbidities of underlying cardiopulmonary disease, cerebrovascular disease, and frailty face an inevitable decline in activity tolerance, which forms a crosstalk loop with physical inactivity. An early study [29] found a 30% decrease in the rate of muscle protein synthesis, a sustained negative nitrogen balance, and a generalized lean body mass loss of approximately 1.50 (− 0.62, − 2.48) kg after 10 days of bed rest in an elderly population with a mean age of 67 ± 5. Resistance exercise induces a cascade response that promotes muscle hypertrophy [20], in which mTORC1 is a key target. MTORC1 stimulates anabolism, reverses negative nitrogen homeostasis, and promotes muscle cell proliferation by initiating the translation of messenger nucleotides from the 0-kDa ribosomal protein S6 kinase (S6 K1) and the eukaryotic initiation factor 4E binding protein-1 (4E-BP1) [30, 31]. Resistance exercise also promotes the ectopic translocation of mTOR to the cell membrane and co-localization with eIF3F at the cell membrane to increase mRNA translation [32].

Routine intake of dietary protein directly affects skeletal muscle protein turnover, promotes muscle damage repair, and improves physical activity performance [33, 34]. High concentrations of amino acids have a direct effect on the rate of muscle synthesis, with one regression study showing that a 100% increase in peripheral EAA concentration resulted in an increase in muscle protein of approximately 34% [35]. One of the essential amino acids, leucine, can reverse the age-related decline of muscle protein synthesis [36, 37].

Long-chain polyunsaturated fatty acids are star anti-inflammatory agents, which not only directly target the anabolic resistance processes associated with aging, but also modulate mTOR activation and increase insulin sensitivity in skeletal muscle [38]. In a model of co-incubation of C2C12 myoblasts with eicosapentaenoic acid (EPA), EPA inhibits impaired LPS-mediated insulin signaling and increased insulin sensitivity and upregulates PPARγ to inhibit the NF-κB-mediated pro-inflammatory microenvironment to activate C2C12 myoblast differentiation [39, 40]. Omega-3 fatty acids accelerate the basal rate of muscle protein formation in the hyperaminoacidemic–hyperinsulinemic clamp, a process accompanied by mTOR activation and S6K1 phosphorylation [41]. N-3 PUFA also reduces muscle loss by attenuating age-induced alterations in vascular function and increasing MyoD and myogenin mRNA [42].

At the same time, social matters (financial abuse, physical abuse and neglect) are strongly associated with sarcopenia and frailty in the elderly, a phenomenon that is more pronounced in the female population. It is therefore necessary to pay more attention to the role of various non-pharmacological interventions, as well as psychological counselling and social assistance, in the prevention and treatment of frailty and sarcopenia in old age [43, 44] .

### Comparison with previous studies

As sarcopenia has gradually come into the public consciousness, related clinical trials have blossomed, and the number of meta-analyses integrating evidence from clinical trials has increased. Lu et al. [9] explored the effects of three exercise modes (resistance training, whole-body vibration training, and mixed training) on muscle strength and physical function in patients with sarcopenia through subgroup analysis, and found that all three modes can decrease TUG completion time, with the first two beings can increase gait speed. Unfortunately, the study did not directly assess the effects of different exercises on muscle mass and only used indirect indicators of muscle condition. Shen et al. [13] recently published a systematic, complete, and in-depth network meta-analysis, comparing the effects of various physical activities on sarcopenia-related indicators (mortality, quality of life, muscle strength, and physical performance indicators), which concluded a remarkable role of resistance exercise in intervening sarcopenia, which is in accordance with our findings. Steffl et al. [45] found that physical activity correlated with preservation of muscle mass, and reduced the risk of sarcopenia by 55%. Chang et al. [46]found that supplementation with whey protein, leucine, and vitamin D increased muscle mass in sarcopenia. However, the trial's unclear diagnostic criteria for sarcopenia, limited number of included trials, and imprecise effect of action may affect the confidence of the outcome.

### Limitations and prospects

To our knowledge, it is the first Bayesian network meta-analysis to synthesize the available evidence for all non-pharmacological interventions to explore effects on muscle mass, physical activity and quality of life for sarcopenia. We used currently prevailing diagnostic criteria for sarcopenia to identify literature for inclusion, instrumentally measured muscle mass as the primary outcome indicator, and meta-regression to identify sources of heterogeneity. This study includes the following limitations. The first is limitation of data, due to the small sample size studies and inevitable global inconsistency which may have an impact on the results. The second is the diagnostic limitation, there are differences in the diagnostic criteria used in this study and the presence of this heterogeneity may have an impact on the results. Therefore, we cannot draw robust conclusions yet.

How to manage the physical activity and nutritional intake of the included elderly subjects is a part that researchers need to study in future related studies. Firstly, unlike fixed drug therapies, a wide range of non-pharmacological interventions face uncertainty and instability in their delivery. Secondly, there are individual differences in the effects of exercise and nutritional interventions. Thirdly, the elderly face the risk of underlying disease and frailty, so how to conduct clinical trials rationally and safely deserves in-depth consideration. Fourthly, the diagnostic criteria for sarcopenia vary greatly in the international arena, and in the process of enrolling subjects, older adults in each region should adopt diagnostic criteria for sarcopenia that are consistent with the general characteristics of that region.

## Conclusion

We reviewed non-pharmacological interventions in sarcopenia and assessed the role of different therapies on outcomes such as muscle mass, physical activity, quality of life, and physical measures in sarcopenia in a Bayesian framework. Based on the combined evidence, we found that resistance exercise (low-moderate load), exercise plus nutrition, and nutritional supplementation (fatty acids, etc.) may be protective against sarcopenia.

Based on these results, we hope that clinical practitioners should be aware of the ameliorative effects of non-pharmacological therapies for sarcopenia.

## Supplementary Information

Below is the link to the electronic supplementary material.

Supplementary file1 (DOCX 60641 KB)

## Data Availability

No datasets were generated or analysed during the current study.
