# Supplementary material for: Seeking optimal non-pharmacological interventions for sarcopenia: a systematic review and network meta-analysis
Source: Aging Clin Exp Res. 2025 Jan 15;37(1):24. doi: 10.1007/s40520-024-02920-6 (PMC11735497; doi:10.1007/s40520-024-02920-6)
Supplement: Supplementary file 1 — Supplementary file1 (DOCX 60641 KB) [file 40520_2024_2920_MOESM1_ESM.docx]

**Supplementary Material**

**Table of Contents**

**Title: Seeking optimal non-pharmacological interventions for sarcopenia: a systematic review and network meta-analysis**

Zhenyue Fu1,2, Pengfei Liu1,2, Lu Zhao1, Yajiao Wang1, Yumeng Li1*, Qingqiao Song1*

^1^ Department of General Internal Medicine, Guang'anmen Hospital, China Academy of Chinese Medical Sciences, Beijing, China

^2^ Beijing University of Chinese Medicine, Beijing, China

*** Correspondence:**

Yumeng Li: lym0707@163.com
Qingqiao Song: songqqbj@126.com

**Directory**

Appendix S1: The PRISMA-NMA checklist of this network meta-analysis. 2

Appendix S2: Search strategy 6

S2.1 Search strategy in PubMed 6

S2.2 Search strategy in Medline OVID 9

S2.3 Search strategy in EMBASE 10

S2.4 Search strategy in Scopus 11

S2.5 Search strategy in Cochrane Medline 11

Appendix S3: The characteristics of included studies. 11

S3.1 Basic information of included studies 11

S3.2 Details of intervention and placebo of included studies 17

S3.3 The number of each intervention category 37

Appendix S4: Risk of bias and Quality evaluation of included studies 38

S4.1 Quality evaluation of included studies based on Corchane Handbook 38

S4.2 Reliability of network meta-analysis primary results based on CINeMA 39

Appendix S5: Consistency assessment 40

S5.1 The PSRF value of each outcome. 40

Appendix S6: Results of SUCRA 42

Appendix S7: Results of Meta-regression 42

S7.1 skeletal muscle mass 42

S7.2 Lean body mass 47

S7.3 The funnel plot of skeletal muscle mass and lean body mass 53

# Appendix S1: The PRISMA-NMA checklist of this network meta-analysis.

| **Section/Topic** | **Item #** | **Checklist Item** | **Reported on Page #** |
| --- | --- | --- | --- |
| **TITLE** |  |  |  |
| Title | 1 | Identify the report as a systematic review *incorporating a network meta-analysis (or related form of meta-analysis).* | 1 |
|  |  |  |  |
| **ABSTRACT** |  |  |  |
| Structured summary | 2 | Provide a structured summary including, as applicable:  **Background:** main objectives  **Methods:** data sources; study eligibility criteria, participants, and interventions; study appraisal; and *synthesis methods, such as network meta-analysis.*  **Results:** number of studies and participants identified; summary estimates with corresponding confidence/credible intervals; *treatment rankings may also be discussed. Authors may choose to summarize pairwise comparisons against a chosen treatment included in their analyses for brevity.*  **Discussion/Conclusions:** limitations; conclusions and implications of findings.  **Other:** primary source of funding; systematic review registration number with registry name. | 1 |
|  |  |  |  |
| **INTRODUCTION** |  |  |  |
| Rationale | 3 | Describe the rationale for the review in the context of what is already known*, including mention of why a network meta-analysis has been conducted.* | ***2*** |
| Objectives | 4 | Provide an explicit statement of questions being addressed, with reference to participants, interventions, comparisons, outcomes, and study design (PICOS). | 2 |
|  |  |  |  |
| **METHODS** |  |  |  |
| Protocol and registration | 5 | Indicate whether a review protocol exists and if and where it can be accessed (e.g., Web address); and, if available, provide registration information, including registration number. | 5 |
| Eligibility criteria | 6 | Specify study characteristics (e.g., PICOS, length of follow-up) and report characteristics (e.g., years considered, language, publication status) used as criteria for eligibility, giving rationale. *Clearly describe eligible treatments included in the treatment network, and note whether any have been clustered or merged into the same node (with justification).* | ***6*** |
| Information sources | 7 | Describe all information sources (e.g., databases with dates of coverage, contact with study authors to identify additional studies) in the search and date last searched. | 5 |
| Search | 8 | Present full electronic search strategy for at least one database, including any limits used, such that it could be repeated. | 5 |
| Study selection | 9 | State the process for selecting studies (i.e., screening, eligibility, included in systematic review, and, if applicable, included in the meta-analysis). | 6 |
| Data collection process | 10 | Describe method of data extraction from reports (e.g., piloted forms, independently, in duplicate) and any processes for obtaining and confirming data from investigators. | 7 |
| Data items | 11 | List and define all variables for which data were sought (e.g., PICOS, funding sources) and any assumptions and simplifications made. | 7 |
| **Geometry of the network** | **S1** | Describe methods used to explore the geometry of the treatment network under study and potential biases related to it. This should include how the evidence base has been graphically summarized for presentation, and what characteristics were compiled and used to describe the evidence base to readers. | ***9*** |
| Risk of bias within individual studies | 12 | Describe methods used for assessing risk of bias of individual studies (including specification of whether this was done at the study or outcome level), and how this information is to be used in any data synthesis. | 8 |
| Summary measures | 13 | State the principal summary measures (e.g., risk ratio, difference in means). *Also describe the use of additional summary measures assessed, such as treatment rankings and surface under the cumulative ranking curve (SUCRA) values, as well as modified approaches used to present summary findings from meta-analyses.* | 9 |
| Planned methods of analysis | 14 | Describe the methods of handling data and combining results of studies for each network meta-analysis. This should include, but not be limited to:   - *Handling of multi-arm trials;* - *Selection of variance structure;* - *Selection of prior distributions in Bayesian analyses; and* - *Assessment of model fit.* | 9 |
| **Assessment of Inconsistency** | **S2** | Describe the statistical methods used to evaluate the agreement of direct and indirect evidence in the treatment network(s) studied. Describe efforts taken to address its presence when found. | 10 |
| Risk of bias across studies | 15 | Specify any assessment of risk of bias that may affect the cumulative evidence (e.g., publication bias, selective reporting within studies). | **10** |
| Additional analyses | 16 | Describe methods of additional analyses if done, indicating which were pre-specified. This may include, but not be limited to, the following:   - Sensitivity or subgroup analyses; - Meta-regression analyses; - *Alternative formulations of the treatment network; and* - *Use of alternative prior distributions for Bayesian analyses (if applicable).* | ***10*** |
|  |  |  |  |
| **RESULTS†** |  |  |  |
| Study selection | 17 | Give numbers of studies screened, assessed for eligibility, and included in the review, with reasons for exclusions at each stage, ideally with a flow diagram. | 11 |
| **Presentation of network structure** | **S3** | Provide a network graph of the included studies to enable visualization of the geometry of the treatment network. | ***14*** |
| **Summary of network geometry** | **S4** | Provide a brief overview of characteristics of the treatment network. This may include commentary on the abundance of trials and randomized patients for the different interventions and pairwise comparisons in the network, gaps of evidence in the treatment network, and potential biases reflected by the network structure. | ***12*** |
| Study characteristics | 18 | For each study, present characteristics for which data were extracted (e.g., study size, PICOS, follow-up period) and provide the citations. | 12 |
| Risk of bias within studies | 19 | Present data on risk of bias of each study and, if available, any outcome level assessment. | 13 |
| Results of individual studies | 20 | For all outcomes considered (benefits or harms), present, for each study: 1) simple summary data for each intervention group, and 2) effect estimates and confidence intervals. *Modified approaches may be needed to deal with information from larger networks.* | ***14*** |
| Synthesis of results | 21 | Present results of each meta-analysis done, including confidence/credible intervals. *In larger networks, authors may focus on comparisons versus a particular comparator (e.g. placebo or standard care), with full findings presented in an appendix. League tables and forest plots may be considered to summarize pairwise comparisons.* If additional summary measures were explored (such as treatment rankings), these should also be presented. | ***14*** |
| **Exploration for inconsistency** | **S5** | Describe results from investigations of inconsistency. This may include such information as measures of model fit to compare consistency and inconsistency models, *P* values from statistical tests, or summary of inconsistency estimates from different parts of the treatment network. | ***25*** |
| Risk of bias across studies | 22 | Present results of any assessment of risk of bias across studies for the evidence base being studied. | 25 |
| Results of additional analyses | 23 | Give results of additional analyses, if done (e.g., sensitivity or subgroup analyses, meta-regression analyses*, alternative network geometries studied, alternative choice of prior distributions for Bayesian analyses,* and so forth). | ***26*** |
|  |  |  |  |
| **DISCUSSION** |  |  |  |
| Summary of evidence | 24 | Summarize the main findings, including the strength of evidence for each main outcome; consider their relevance to key groups (e.g., healthcare providers, users, and policy-makers). | 26 |
| Limitations | 25 | Discuss limitations at study and outcome level (e.g., risk of bias), and at review level (e.g., incomplete retrieval of identified research, reporting bias). *Comment on the validity of the assumptions, such as transitivity and consistency. Comment on any concerns regarding network geometry (e.g., avoidance of certain comparisons).* | 29 |
| Conclusions | 26 | Provide a general interpretation of the results in the context of other evidence, and implications for future research. | 30 |
|  |  |  |  |
| **FUNDING** |  |  |  |
| Funding | 27 | Describe sources of funding for the systematic review and other support (e.g., supply of data); role of funders for the systematic review. This should also include information regarding whether funding has been received from manufacturers of treatments in the network and/or whether some of the authors are content experts with professional conflicts of interest that could affect use of treatments in the network. | ***31*** |

PICOS = population, intervention, comparators, outcomes, study design.

* Text in italics indicateS wording specific to reporting of network meta-analyses that has been added to guidance from the PRISMA statement.

# Appendix S2: Search strategy

Time setting: from 2000.1.1 to 2023.10.27

Language setting: English

Database: PubMed、Medline OVID、EMBASE、Scopus、Cochrane Medline

## S2.1 Search strategy in PubMed

| Number | Query | Results |
| --- | --- | --- |
| 1 | "Sarcopenia"[Mesh] | 9,670 |
| 2 | Sarcopenias[Title/Abstract] | 4 |
| 3 | Muscular Atrophy[Title/Abstract] | 10,502 |
| 4 | Sarcopeni*[Title/Abstract] | 17,383 |
| 5 | Myopeni*[Title/Abstract] | 79 |
| 6 | ((((Muscle loss[Title/Abstract]) OR (Muscle depletion[Title/Abstract])) OR (Muscle wasting[Title/Abstract])) OR (Muscle reduction[Title/Abstract])) OR (Muscle attenuation[Title/Abstract]) | 9,293 |
| 7 | (((((fat free mass[Title/Abstract]) OR (lean mass[Title/Abstract])) OR (lean mass[Title/Abstract])) OR (lean mass[Title/Abstract])) OR (handgrip strength[Title/Abstract])) OR (grip strength[Title/Abstract]) | 36,919 |
| 8 | (((((((((((fat free mass[Title/Abstract]) OR (lean mass[Title/Abstract])) OR (lean mass[Title/Abstract])) OR (lean mass[Title/Abstract])) OR (handgrip strength[Title/Abstract])) OR (grip strength[Title/Abstract])) OR (((((Muscle loss[Title/Abstract]) OR (Muscle depletion[Title/Abstract])) OR (Muscle wasting[Title/Abstract])) OR (Muscle reduction[Title/Abstract])) OR (Muscle attenuation[Title/Abstract]))) OR (Myopeni*[Title/Abstract])) OR (Sarcopeni*[Title/Abstract])) OR (Muscular Atrophy[Title/Abstract])) OR (Sarcopenias[Title/Abstract])) OR ("Sarcopenia"[Mesh]) | 68,335 |
| 9 | (randomized controlled trial[pt] OR controlled clinical trial[pt] OR randomized[tiab] OR randomly[tiab] OR trial[tiab]) NOT (animals[mh] NOT humans[mh]) | 1,513,053 |
| 10 | ((randomized controlled trial[pt] OR controlled clinical trial[pt] OR randomized[tiab] OR randomly[tiab] OR trial[tiab]) NOT (animals[mh] NOT humans[mh])) AND ((((((((((((fat free mass[Title/Abstract]) OR (lean mass[Title/Abstract])) OR (lean mass[Title/Abstract])) OR (lean mass[Title/Abstract])) OR (handgrip strength[Title/Abstract])) OR (grip strength[Title/Abstract])) OR (((((Muscle loss[Title/Abstract]) OR (Muscle depletion[Title/Abstract])) OR (Muscle wasting[Title/Abstract])) OR (Muscle reduction[Title/Abstract])) OR (Muscle attenuation[Title/Abstract]))) OR (Myopeni*[Title/Abstract])) OR (Sarcopeni*[Title/Abstract])) OR (Muscular Atrophy[Title/Abstract])) OR (Sarcopenias[Title/Abstract])) OR ("Sarcopenia"[Mesh])) | 8,463 |
| 11 | (randomized controlled trial[pt] OR controlled clinical trial[pt] OR randomized[tiab] OR placebo[tiab] OR clinical trials as topic[mesh:noexp] OR randomly[tiab] OR trial[ti]) NOT (animals[mh] NOT (humans[mh] AND animals[mh])) | 1,446,622 |
| 12 | ((randomized controlled trial[pt] OR controlled clinical trial[pt] OR randomized[tiab] OR placebo[tiab] OR clinical trials as topic[mesh:noexp] OR randomly[tiab] OR trial[ti]) NOT (animals[mh] NOT (humans[mh] AND animals[mh]))) AND ((((((((((((fat free mass[Title/Abstract]) OR (lean mass[Title/Abstract])) OR (lean mass[Title/Abstract])) OR (lean mass[Title/Abstract])) OR (handgrip strength[Title/Abstract])) OR (grip strength[Title/Abstract])) OR (((((Muscle loss[Title/Abstract]) OR (Muscle depletion[Title/Abstract])) OR (Muscle wasting[Title/Abstract])) OR (Muscle reduction[Title/Abstract])) OR (Muscle attenuation[Title/Abstract]))) OR (Myopeni*[Title/Abstract])) OR (Sarcopeni*[Title/Abstract])) OR (Muscular Atrophy[Title/Abstract])) OR (Sarcopenias[Title/Abstract])) OR ("Sarcopenia"[Mesh])) | 7,713 |

## S2.2 Search strategy in Medline OVID

1 ((randomized controlled trial or controlled clinical trial).pt. or randomized.ab. or placebo.ab. or clinical trials as topic.sh. or randomly.ab. or trial.ti.) not (exp animals/ not humans.sh.) 1432672

2 exp Sarcopenia/ 9673

3 "Sarcopeni*".ab,ti. 15215

4 "Myopeni*".ab,ti. 73

5 Muscle loss.ab,ti. 2796

6 Muscle depletion.ab,ti. 227

7 Muscle wasting.ab,ti. 6053

8 Muscle reduction.ab,ti. 71

9 muscle attenuation.ab,ti. 285

10 fat free mass.ab,ti. 9409

11 lean mass.ab,ti. 7384

12 muscle fatigue.ab,ti. 5260

13 muscle weakness.ab,ti. 18006

14 handgrip strength.ab,ti. 5621

15 handgrip strength.ab,ti. 5621

16 2 or 3 or 4 or 5 or 6 or 7 or 8 or 9 or 10 or 11 or 12 or 13 or 14 or 15 65024

17 1 and 16 6361

18 17 and 2000:2024.(sa_year). 5935

## S2.3 Search strategy in EMBASE

| No. | Query | Results |
| --- | --- | --- |
| 1 | sarcopenia:de,ab,ti | 26381 |
| 2 | sarcopeni*:de,ab,ti | 27317 |
| 3 | myopeni*:de,ab,ti | 143 |
| 4 | 'muscle loss':de,ab,ti | 4154 |
| 5 | 'muscle depletion':de,ab,ti | 397 |
| 6 | 'muscle atrophy':de,ab,ti | 46463 |
| 7 | 'muscle attenuation':de,ab,ti | 473 |
| 8 | 'fat free mass':de,ab,ti | 14414 |
| 9 | 'lean mass':de,ab,ti | 11496 |
| 10 | 'muscle weakness':de,ab,ti | 69369 |
| 11 | 'grip strength':de,ab,ti | 40733 |
| 12 | #1 OR #2 OR #3 OR #4 OR #5 OR #6 OR #7 OR #8 OR #9 OR #10 OR #11 | 189502 |
| 13 | 'crossover procedure':de OR 'double-blind procedure':de OR 'randomized controlled trial':de OR random*:de,ab,ti OR placebo*:de,ab,ti | 2501652 |
| 14 | #12 AND #13 | 20203 |

## S2.4 Search strategy in Scopus

( TITLE-ABS-KEY ( "Sarcopenias" OR "Sarcopeni*" OR "Myopeni*" OR "Muscle loss" OR "Muscle depletion" OR "Muscle wasting" OR "Muscle reduction" OR "muscle attenuation" OR "fat free mass" OR "lean mass" OR "muscle fatigue" OR "muscle weakness" OR "handgrip strength" OR "grip strength" ) ) AND ( ( INDEXTERMS ( "clinical trials" OR "clinical trials as a topic" OR "randomized controlled trial" OR "Randomized Controlled Trials as Topic" OR "controlled clinical trial" OR "Controlled Clinical Trials" OR "random allocation" OR "Double-Blind Method" OR "Single-Blind Method" OR "Cross-Over Studies" OR "Placebos" OR "multicenter study" OR "double blind procedure" OR "single blind procedure" OR "crossover procedure" OR "clinical trial" ) ) ) AND ( LIMIT-TO ( LANGUAGE , "English" ) )

## S2.5 Search strategy in Cochrane Medline

1# (“Sarcopenias”OR“Muscle loss”OR“Muscle depletion”OR“Muscle wasting”OR“Muscle reduction”OR“muscle attenuation”OR“fat free mass”OR“lean mass”OR“muscle fatigue”OR“muscle weakness”OR“handgrip strength”OR“grip strength”):ti,ab,kw

2# ("randomized controlled trial"OR"controlled clinical trial"OR"randomized"OR"randomly"OR"trial"):ti,ab,kw

3# 1 AND 2

# Appendix S3: The characteristics of included studies.

## S3.1 Basic information of included studies

| First author/Year | Country | Trial registration number | Design | Funding | Diagnostic criteria | ITT | setting | Center sites | Total  sample | Mean age | | BMI | | Male% | baseline disease | duration |
| --- | --- | --- | --- | --- | --- | --- | --- | --- | --- | --- | --- | --- | --- | --- | --- | --- |
|  |  |  | 1=parallel 2=crossover 3=cluster | 1=industry 2=government 3=institution 0=none | 1=EWGSOP1/2 2=AWGS 3=other | 1=yes 0=no | 1=Community 2=Hospital/Institution 3=Nursing home 4=other/NP | 1=single center 2=multi center 3=NP |  | **T** | **C** |  |  |  |  |  |
| Alemán-Mateo H. 2012[1] | México | NP | 1 | 1,2 | 3 ALM/ht2: men<7.25 kg/ht2 women<5.67 kg/ht2 | 0 | 4 | 1 | 29 | 76 (5.4) | | 26.3(3.8) | | 42.5% | non | 3m |
| Bauer, JM. 2015[2] | Europe | NTR2329 | 1 | 1 | 3 SMI: men≤37%, women≤28% | 1 | 4 | 2 | 380 | 77.3 (6.7) | 78.1 (7.0) | 26.0 (2.5) | 26.2 (2.8) | 34.47% | non | 13w |
| Bernabei, R. 2022[3] | Italy | NCT02582138 | 1 | 1,2,3 | 3 FNIH | 1 | 1 | 2 | 1205 | 79.09 (5.87) | 78.77 (5.78) | 28.60 (5.44) | 28.62 (5.94) | 28.71% | frailty | 36m |
| Björkman, MP. 2020[4] | Finland | ACTRN12612001253897 | 1 | 2,3 | 3 HGS: men ≤30.0 kg, women ≤20.0 kg GS ≤0.80 m/s CRi-SMI: men<2.06 cm 2/Ω, women1.50 cm 2/Ω | 0 | 1 | 1 | 146 | 84.0 (3.9) | 83.6 (4.7) | NP | | 34.25% | non | 12m |
| Bo, CY. 2019[5] | China | ChiCTR-IOR-16008155 | 1 | 1 | 3 RSMI: women<5.7 kg/m2, men<7.0 kg/m2  HGS: women<18 kg, men<26 kg 6-m usual walk speed<0.8 m/s | 0 | 4 | 1 | 60 | 74.83 (5.94) | 73.23 (6.52) | NP | | 45.00% | non | 6m |
| Chen, BY. 2023[6] | China | NP | 1 | 2,3 | 2 | 1 | 1 | 1 | 60 | 65.68 (2.5) | 65.21 (2.6) | NP | | 0.00% | non | 8m |
| Chen, HT. 2017[7] | Taiwan, China | NP | 1 | 2 | 3 ASM/Weight: men≤32.5%, women≤25.7% | 0 | 1,2 | 1 | 60 | 68.6 (3.1) | | NP | | 16.67% | sarcopenic obesity | 8w |
| Chen, HT. 2018[8] | Taiwan, China | NP | 1 | 0 | 2 | 0 | 1,2 | 1 | 33 | 66.7 (5.3) | | NP | | 100.00% | non | 12w |
| Cramer, J. 2015[9] | Italy | NCT01191125 | 1 | 2 | 1 | 0 | 4 | 2 | 184 | 77 (71, 81) | | NP | | NP | malnutrition | 6m |
| Ferhi, H. 2023[10] | Tunis | PACTR202306912191110 | 1 | 0 | 3 HGS < 17 N GS < 1.0 m/s | 0 | 2 | 2 | 40 | 74.1 (3.7) | 76.6 (5.6) | 35.8 (2.7) | 35.8 (2.7) | NP | sarcopenic obesity | 6m |
| Flor-Rufino, C. 2023[11] | Spain | NCT03834558 | 1 | 3 | 1 | 0 | 1,2 | 3 | 38 | 79.8 (7.4) | | NP | | NP | non | 6m |
| Huang, SW. 2017[12] | Taiwan, China | ChiCTR-IPR-15006069 | 1 | 3 | 3 SMI: women<27.6% | 0 | 1,2 | 3 | 35 | 68.89 (4.91) | 69.53 (5.09) | 27.31 (3.74) | 28.96 (3.49) | NP | sarcopenic obesity | 12w |
| Jung, WS. 2019[13] | Korea | NP | 1 | 3 | 3 ASM/h2<5.4 | 0 | 1 | 1 | 26 | 75 (3.9) | 74.9 (5.2) | 21.8 (1.5) | 21.9 (1.5) | NP | non | 12w |
| Kemmler, W. 2018[14] | German | NCT02857660 | 1 | 0 | 3 SMI<5.75kg/m2 | 1 | 1 | 2 | 67 | 77.1 ( 4.3) | 76.9 (5.1) | NP | | NP | sarcopenic obesity | 16w |
| Kemmler, W. 2020[15] | German | NP | 1 | 3 | 3 SMI<7.26kg/m2 | 1 | 1 | 2 | 43 | 77.8 (3.6) | 79.2 (4.7) | 25.0 (3.0) | 24.5 (1.9) | NP | Osteosarcopenia | 18m |
| Kemmler, W. 2016[16] | German | NCT02356016 | 1 | 0 | 3 SMI<5.75kg/m2 | 1 | 1 | 2 | 75 | 77.3 (4.9) 76.4 (2.9) | 77.4 (4.9) | NP | | NP | sarcopenic obesity | 16w |
| Kim, H. 2016[17] | Japan | NP | 1 | 1,2 | 3 SMI<5.67 kg/m2 HGS<17.0 kg walking speed<1.0 m/s. | 0 | 1 | 2 | 139 | 80.9 (4.2)  81.4 (4.3)  81.2 (4.9) | 81.1 (5.1) | 24.9 (3.0)  25.1 (2.5)  24.9 (2.5) | 25.3 (2.8) | 0.00% | sarcopenic obesity | 3m |
| Kim, H. 2013[18] | Japan | NP | 1 | 2 | 3 (i)ASM/h2<6.42 kg/m2, knee extension strength<1.01 Nm/kg  (ii)ASM/h2<6.42 kg/m2, walking speed<1.10 m/sec  (iii)BMI<22, knee extension strength<1.10 Nm/kg (iv)BMI<22, walking speed<1.10 m/sec | 0 | 1 | 2 | 128 | 81.1 (3.7) 79.6 (4.2) 80.0 (4.0) | 80.2 (5.6) | NP | | NP | non | 3m |
| Kim, H. 2012[19] | Japan | NP | 1 | 2 | 3 ASM/h2<6.42 kg/m2, knee extension strength<1.01 Nm/kg ASM/h2<6.42 kg/m2, usual walking speed<1.22 m/s  BMI<22.0 kg/m2, knee extension strength<1.01 Nm/kg BMI<22.0 kg/m2, usual walking speed<1.22 m/s | 0 | 1 | 2 | 155 | 79.5 (2.9) 79.0 (2.9) 79.2 (2.8) | 78.7 (2.8) | 18.3 (2.5) 18.9 (2.0) 18.9 (1.6) | 18.8 (1.7) | NP | non | 3m |
| Kwon, IS. 2021[20] | Korea | NP | 1 | 1 | 3 FNIH | 0 | 4 | 1 | 20 | 74.63 (9.30) | 73.38 (10.66) | 33.76 (9.38) | 25.39 (4.46) | NP | non | 4w |
| Lee, YH. 2021[21] | Taiwan, China | ChiCTR-IPR-15006069 | 1 | 2,3 | 1 | 0 | 1 | 1 | 27 | 70.13 (4.41) | 71.82 (5.23) | 26.95 (3.31) | 28.93 (3.55) | NP | osteosarcopenic adiposity | 12w |
| Li, C. 2021[22] | China | NCT02490410 | 1 | 2 | 3 ASMI men< 7.0 kg/m2 women< 5.4 kg/m2 | 1 | 1 | 3 | 123 | 71 (4)  69 (4)  70 (4) | 71 (4) | 21.8 (2.0) 21.2 (2.3) 20.6 (1.8) | 20.8 (2.2) | 49.59% | non | 6m |
| Li, Z. 2021[23] | China | NCT02873676 | 1 | 2 | 2 | 1 | 1 | 2 | 241 | 71.52 (5.28) 73.73 (5.69) 70.04 (3.98) | 72.91 (6.29) | 23.5 (3.26) 23.29 (2.83) 22.87 (2.2) | 22.63 (2.89) | 29.05% | non | 12w |
| Liao, CD. 2018[24] | Taiwan, China | ChiCTR-IPR-15006069 | 1 | 2,3 | 3 SMI<27.6% in women | 1 | 4 | 3 | 56 | 66.67 (4.54) | 68.32 (6.05) | 27.27 (3.72) | 29.16 (3.62) | NP | non | 12w |
| Lin, CC. 2021[25] | Taiwan, China | NCT03860194 | 1 | 0 | 2 | 0 | 2 | 1 | 85 | 72.5 (5.57) | 73.8 (8.11) | 20.6 (1.9) | 19.8 (1.8) | 71.43% | non | 12w |
| Moghadam, BH. 2020[26] | HK, China | NP | 1 | 0 | 3 HGS <26–30 kg, walking speed<0.8 m/s SMI of less than two standard deviations below the average young adult population | 0 | 4 | 3 | 30 | 64.3(3.5) | | 22.6 (1.1) | 22.9 (1.3) | NP | non | 8w |
| Nasimi, N. 2021[27] | Iran | IRCT20171223038017N1 | 1 | 3 | 2 | 0 | 1 | 2 | 66 | 71 (3.3) | 69 (7) | 23.02 (3.88) | 23.30 (2.44) | 75.76% | non | 12w |
| Ning, W. 2017[28] | HK, China | NP | 1 | 2 | 3 SMI male<8.87kg/m2 female <6.42kg/m2 | 1 | 1 | 1 | 40 | 75 (6) | 76 (6) | 23.44 (2.47) | 23.83 (2.65) | 30.00% | non | 12w |
| Rondanelli, M. 2022[29] | Italy | NCT04702087 | 1 | 1 | 1 | 0 | 2 | 1 | 60 | 78.84 (5.80) | | 22.63 (2.74) | 21.96 (1.34) | NP | non | 2m |
| Rondanelli, M. 2018[30] | Italy | NCT03784495 | 1 | 0 | 3 SMI  men <7.23 kg/m2 women<5.45 kg/m2 | 0 | 2 | 1 | 159 | 81.64 (7.04) 80.55 (6.76) 81.42 (8.02) | 81.86 (6.43) | 24.04 (0.83) 21.94 (0.72) 24.29 (0.67) | 22.86 (0.63) | 26.42% | non | 4w |
| Sammarco, R. 2017[31] | Italy | NP | 1 | 2 | 3 Fat mass > 34.8% FFM was considered depleted if it was <90% of subject's ideal FFM | 0 | 2 | 1 | 18 | 58 (10) | 53 (8.9) | NP | | NP | sarcopenic obesity | 4m |
| Sen, EI. 2021[32] | Turkey | NCT04598464 | 1 | 0 | 3 SARC-F ≥4 GS ≤0.8 m/s SPPB ≤ | 0 | 2 | 2 | 100 | 73(4.8) | 72.7(5) | 28.7(4.8) | 28.7(4.6) | NP | non | 3m |
| Seo, MW. 2021[33] | Korea | NP | 1 | 2 | 1 | 0 | 4 | 3 | 22 | 70.3 ( 5.38 | 72.9 ( 4.75 | 22.9 ( 2.02 | 22.4 ( 1.52 | NP | non | 16w |
| Shahar, S. 2013[34] | Malaysia | NP | 1 | 2 | 3 skeletal muscle cutoff points  men<10.75 kg/m2 women<6.75 kg/m2 | 0 | 1 | 1 | 65 | 69.74(5.46) 65.93(4.37) 65.20(4.87) | 67.25(5.48) | 23.71(3.46) 24.26(4.37) 26.47(4.75) | 26.36(3.19) | NP | non | 12w |
| Soares Mendes  Damasceno, G.  2019[35] | Brazil | RBR-8df2h4 | 1 | 0 | 1   BIA: men: 8.87 kg/m2; women: 6.42 kg/m2   Handgrip: men: <30 kg; women: <20 kg   TUG: >0.8 M/S | 0 | 1 | 1 | 15 | 72(7.9) | 63.5(3.3) | NP | | NP | non | 8w |
| Tamura, Y. 2023[36] | Japan | jRCTs031200433 | 1 | 1 | 3 walking speed<1 m/s HGS: men<25 kg; women<20 kg BMI<18.5 kg/m2 lower leg circumference: men<34cm; women<33cm SMI: men<7.0 kg/m2; women<5.7 kg/m2 | 1 | 2 | 2 | 35 | 80.3 (8.3) | 82.1 (6.1) | NP | | 22.86% | non | 12w |
| Tsekoura, M. 2018[37] | Greece | ISRCTN92538100 | 1 | 0 | 1 | 0 | 2,3 | 2 | 36 | 74.56 (6.04) | 72.89 (8.31) | 21.95 ( 2.18 | 22.98 ( 2.29 | 11.11% | non | 12w |
| Vasconcelos, KS.  2016[38] | Brazil | NP | 1 | 1 | 3 BMI ≥30 kg/m2  HGS≤21 kg | 0 | 1 | 3 | 28 | 72 (4.6) | 72 (3.6) | 32 (2.3) | 33 (2.9) | NP | sarcopenic obesity | 10w |
| Vezzoli, A. 2019[39] | italy | NP | 1 | 1 | 3 SMI: men<37%; women<28% | 0 | 1 | 3 | 35 | 73.0 (5.5) | 71.7 (3.4) | 27.7 (4.4) | 26.6 (3.5) | NP | non | 12w |
| Wang, R. 2020[40] | China | NP | 1 | 2 | 2 | 0 | 2 | 1 | 121 | 88.2 (5.0) | 87.4 (4.8) | 22.2 (3.7) | 21.6 (3.5) | 77.69% | non | 2w |
| Wang, Z. 2022[41] | China | ChiCTR2100048874 | 1 | 0 | 1+2 | 0 | 1 | 2 |  | 70.16 (4.32) 68.18 (3.93) 69.72 (3.60) | 69.88 (3.29) | 23.68 (3.87) 22.26 (2.4) 23.15 (2.89) | 22.67 (3.07) | NP | non | 12w |
| Wei, M. 2022[42] | China | NP | 1 | 2 | 2 | 0 | 2 | 2 | 90 | 66.70 (4.10) 66.87 (3.84) | 65.42 (3.97) | 22.76 (2.19) 22.80 (3.18) | 21.93 (2.86) | NP | non | 24w |
| Xiao, Y. 2023[43] | China | ChiCTR2100051727 | 1 | 2 | 2 | 0 | 2 | 1 | 60 | 53.9 (14.5) | 51.2 (12.9) | 17.2 (2.2) | 17.0 (2.3) | 50.00% | intestinal failure | 4w |
| Yamada, M. 2019[44] | Japan | NP | 1 | 2 | 2 | 1 | 1 | 3 | 112 | 84.9 (5.6) 84.7 (5.1) 83.2 (5.7) | 83.9 (5.7) | 21.3 (3.2) 22.6 (3.0) 22.6 (4.2) | 21.2 (2.9) | 65.18% | non | 12w |
| Yin, YH. 2023[45] | China | NCT04690985 | 1 | 3 | 2 | 1 | 1 | 2 | 60 | 68.87 (6.51) | 67.40 (5.72) | NP | | 30.00% | non | 15w |
| Yuenyongchaiwat, K. 2022[46] | Thailand | TCTR20201023002 | 1 | 0 | 2 | 1 | 1 | 3 | 60 | 69.23 (6.71) | 71.93 (5.19) | NP | | 26.67% | non | 12w |
| Zhu, Y. 2019[47] | China | NP | 1 | 2 | 2 | 0 | 2 | 1 | 79 | 88.8 (3.7) 89.5 (4.4) | 87.5 (3.0) | 22.4 (3.1) 22.5 (2.2) | 23.5 (3.9) | NP | non | 8w |

Note: ALMI=Appendix Lean Mass Index, SMI=skeletal muscle mass, HGS=handgrip muscle mass, GS=gait speed, NP=not present

## S3.2 Details of intervention and placebo of included studies

| **First author Year** | **Intervention** | **Placebo** | | **Intervention 1** | | | **Intervention 2** | | | **Intervention 3** | | | **Outcome** |
| --- | --- | --- | --- | --- | --- | --- | --- | --- | --- | --- | --- | --- | --- |
|  | 1=RLML, 2=rRHL 3=AE, 4=CE 5=WBVE, 6=PT 7=VT, 8=ON 9=PplusV, 10=EplusN 13=AC, 14=MN | **Placebo description (dose,duration)** | **N** | **Intervention 1  name** | **Intervention 1 description(dose,duration)** | **N** | **Intervention 2  name** | **Intervention 2  description(dose,duration)** | **N** | **Intervention 3  name** | **Intervention 3 description(dose,duration)** | **N** |  |
| Alemán-Mateo H. 2012 | 8 | habitual diet 3mo | 17 | ricotta cheese plus the habitual diet | ricotta cheese (15.7 g of protein, 18.4 g of fat, and 10.4 g of carbohydrates) 210 g/day  3mo | 12 |  |  |  |  |  |  | body weight, LBM, TSM, hand grip |
| Bauer, JM. 2015 | 9 | iso-caloric control product 40g/day 13w | 196 | vitamin D and leucine-enriched whey protein nutritional supplement | 20 g whey protein, 3 g total leucine, 9 g carbohydrates, 3 g fat, 800 IU vitamin D, and a mixture of vitamins, minerals, and fibers 40g/day 13w | 184 |  |  |  |  |  |  | hand grip, chair standing, gait speed |
| Bernabei, R. 2022 | 4 | education once/m 36w | 600 | multicomponent intervention |  | 605 |  |  |  |  |  |  | LBM, hand grip |
| Björkman, MP. 2020 | 6 | isocaloric placebo 20% whey of extra milk derived proteins daily 7.5g/d 12m | 73 | Protein Supplementation | 48% whey of extra milk derived proteins daily 40g/day 12m | 73 |  |  |  |  |  |  | body weight |
| Bo, CY. 2019 | 9 | isocaloric placebo Carbohydrates 84.7 %, Fat 15.3%, Carbohydrates 32.4 g, Fat 2.6g 80g/day 6m | 30 | high whey protein, vitamin D and E supplement | Protein 57.5 %,Carbohydrates 27.2 %, Fat 15.3 %, Protein 22 g,Carbohydrates 10.4 g,Fat 2.6 g,Vitamin D 702 IU,Vitamin E 109 mg 80g/day 6m | 30 |  |  |  |  |  |  | body weight, BMI, LBM, hand grip, chair standing, gait speed |
| Chen, BY. 2023 | 4 | health public education once/2w | 30 | moderate-intensity comprehensive exercise training program | Simplified 24-form Tai Chi exercise plus Progressive resistance exercise  3 times per week 8m | 30 |  |  |  |  |  |  | LBM, TSM, hand grip, chair standing, gait speed |
| Chen, HT. 2017 | 1,3,4 | daily lifestyles | 15 | resistance training | 60min training on weight-training equipment at 60–70% of one repetition maximum once/48h 8w | 15 | aerobic training | 5–10 minutes of dynamic stretching and warm up + 40–45 minutes of the actual training(stepping on the spot, knee lifts, high knee running, rowing arm swings, arm swings, twist steps, arm raises, squats, V steps, mambo steps, diamond steps, and point step jumps) + 10 minutes of closing and relaxation exercises twice/w 8w | 15 | resistance training+aerobic training | performed each training mode once a week with the AT following 48 hours after the RT once/w 8w | 15 | body weight, BMI, LBM, TSM, hand grip |
| Chen, HT. 2018 | 1 | daily lifestyles 8w | 16 | kettlebell training | 60min kettlebell training(kettlebell weight training with 60%–70% of 1 repetition maximum (RM)) twice/w 8w | 17 |  |  |  |  |  |  | body weight, LBM, TSM, hand grip |
| Cramer, J. 2015 | 9 | isocaloric placebo (contained 14 g protein, 11 g fat, 44 g carbohydrate, 147 IU vitamin D 3 , and additional vitamins and minerals.) 24w | 101 | high-quality oral nutritional supplements | E ONS (provided 20 g protein, 11 g fat, 36 g carbohydrate, 1.5 g CaHMB, 499 IU vitamin D 3 , and other vitamins, minerals, and nutrients in varying amounts) 440ml/day 6m | 83 |  |  |  |  |  |  | body weight, BMI, TSM, hand grip, gait speed |
| Ferhi, H. 2023 | 4 | control group (CG) | 20 | posture, strengthening, and motricity (PSM) program | includes a warm-up (10 min), motor skill exercises (duration based on the pre-set training volume), strengthening/posture exercises (duration based on the pre-set training volume), and a cool-down phase (5 min) twice/w 24w | 20 |  |  |  |  |  |  | BMI, LBM, hand grip, gait speed |
| Flor-Rufino, C. 2023 | 2 | control group (CG) | 18 | HIRT | 10-minute warm up+45-min HIRT circuit+10~15-min repetitions twice/W 6m | 20 |  |  |  |  |  |  | body weight, BMI, TSM, hand grip, gait speed |
| Huang, SW. 2017 | 1 | 40-min lesson about SO and home exercise concept 12W | 17 | progressive elastic band resistance training | 10min warm-up+45min elastic band resistance exercise +5min cooling down 3 times/w 12w | 18 |  |  |  |  |  |  | TSM |
| Jung, WS. 2019 | 2 | daily lifestyles 12w | 13 | circuit training exercise | circuit training exercise(walking in place, shoulder press and squat, twist dash. Lunge, jumpng jacks, kick back, push up, crunch, hip bridge, and bird dog) intensity levels ranged from 60%-80% of the heart rate reserve 3 times/w 12w | 13 |  |  |  |  |  |  | body weight, BMI, LBM, gait speed |
| Kemmler, W. 2018 | 10 | control group (CG) Vitamin-D Supplementation 800 IU/day | 34 | whole-body electromyostimulation (WB-EMS) and protein supplements | whey protein powder(1.7–1.8 g/kg/day body mass) 20min impulse WB-EMS Intervention(frequency of 85 Hz, an impulse width of 350 µs, and applied an interval approach with 4 s of stimulation and 4 s of rest) 1.5times/w 16w Vitamin-D Supplementation 800 IU/day | 33 |  |  |  |  |  |  | LBM, gait speed |
| Kemmler, W. 2020 | 10 | daily physical activity/exercise habits Protein Supplementation(1.2–1.3 g/kg body mass/day) Vitamin D and calcium supplementation | 22 | high-velocity/intensity/effort progressive resistance training (HIT-RT) | Protein Supplementation(1.5–1.6 g/kg/day) Vitamin D and calcium supplementation periodized HIT-RT with intensifying strategies (however without MF), varying relative (60–85% 1RM) and absolute (nRM to RM) exercise intensities, and alternating movement velocity (explosive-slow)/time under load/rep (3–9 s) 3 times/w 18m | 21 |  |  |  |  |  |  | LBM |
| Kemmler, W. 2016 | 5,10 | control group (CG) Vitamin-D Supplementation 800 IU/day | 25 | whole-body electromyostimulation (WB-EMS) | 20min impulse WB-EMS Intervention(frequency of 85 Hz, an impulse width of 350 µs,intermittently with 4–6 s of EMS simulation using a direct impulse boost and 4 s of rest) 1.5times/w 16w Vitamin-D Supplementation 800 IU/day | 25 | whole-body electromyostimulation (WB-EMS) and protein supplements | whey protein powder(40 g/day body, contained 21 g of (whey) protein, leucine/L-leucine (2.8 g/portion), essential amino acid (27 g) component, 7 % of fat, 24 % of carbohydrates, 3 % of fibers and 500 mg of calcium) 20min impulse WB-EMS Intervention(frequency of 85 Hz, an impulse width of 350 µs, intermittently with 4–6 s of EMS simulation using a direct impulse boost and 4 s of rest) 1.5times/w 16w Vitamin-D Supplementation 800 IU/day | 25 |  |  |  | hand grip, gait speed |
| Kim, H. 2016 | 4,6,10 | control group Healthy education once/2w 3m | 34 | Exercise+Nutrition | 60min Resistance and weight-bearing exercise(Chair exercise, Resistance band exercise, Hydraulic exercise machine)+Aerobic training(stationary bicycle for 12 minutes, including 1 minute of cooldown, starting at 40 watts) twice/w 3m Amino acid supplementation(3.0 g of leucine-enriched essential amino acid, 20 μg vitamin D)+Tea catechin(350 mL of tea fortified with 540 mg of catechin) everyday 3m | 36 | Exercise | 60min Resistance and weight-bearing exercise(Chair exercise, Resistance band exercise, Hydraulic exercise machine)+Aerobic training(stationary bicycle for 12 minutes, including 1 minute of cooldown, starting at 40 watts) twice/w 3m | 35 | Nutrition | Amino acid supplementation(3.0 g of leucine-enriched essential amino acid, 20 μg vitamin D)+Tea catechin(350 mL of tea fortified with 540 mg of catechin) everyday 3m | 34 | LBM, hand grip, gait speed |
| Kim, H. 2013 | 4,8,10 | control group Healthy education once/m 3m | 32 | Exercise+Tea catechin | 60min exercise(5-min stretching warm-up, 30-min of strengthening exercises, 20 min of balance and gait training, followed by a 5-min cool-downstretching), including muscle strengthening, balance and gait training of moderate intensity twice/w 3m Tea catechin(350 mL of tea fortified with 540 mg of catechin) everyday 3m | 32 | Exercise | 60min exercise(5-min stretching warm-up, 30-min of strengthening exercises, 20-min of balance and gait training, followed by a 5-min cool-downstretching), including muscle strengthening, balance and gait training of moderate intensity twice/w 3m | 32 | Tea catechin | Tea catechin(350 mL of tea fortified with 540 mg of catechin) everyday 3m | 32 | LBM, TSM, hand grip, gait speed, TUG |
| Kim, H. 2012 | 4,6,10 | control group Healthy education once/m 3m | 39 | Exercise+Amino Acid | 60min exercise(5-min stretching warm-up, 30-min of strengthening exercises, 20-min of balance and gait training, followed by a 5-min cool-downstretching), including Chair exercise, Ankle-weight exercise, Exercises using a resistance band, Balance and gait training twice/w 3m 6g amino acid supplements (42.0% leucine, 14.0% lysine, 10.5% valine, 10.5% isoleucine, 10.5% threonine, 7.0% phenylalanine, and 5.5% other) everyday 3m | 38 | Exercise | 60min exercise(5-min stretching warm-up, 30-min of strengthening exercises, 20-min of balance and gait training, followed by a 5-min cool-downstretching), including Chair exercise, Ankle-weight exercise, Exercises using a resistance band, Balance and gait training twice/w 3m | 39 | Amino Acid | 6g amino acid supplements (42.0% leucine, 14.0% lysine, 10.5% valine, 10.5% isoleucine, 10.5% threonine, 7.0% phenylalanine, and 5.5% other) everyday 3m | 39 | BMI, LBM, TSM, gait speed |
| Kwon, IS. 2021 | 6 | placebo dextrin with similar flavor and taste everyday 4w | 10 | marine oligomeric polyphenol (MOP) | 0.7g Mannas powder products(consist of 99% dextrin + 1% MOP)+500ml water everyday 4w | 10 |  |  |  |  |  |  | BMI, LBM, TSM, hand grip, TUG, gait speed |
| Lee, YH. 2021 | 1 | 40-min group lecture | 12 | progressive elastic band resistance exercise | consisted of 10 min of warm-up exercises followed by 40 min of elastic band resistance exercises and 5 min of cooling-down exercises 3 times/w 3m | 15 |  |  |  |  |  |  | LBM, TSM, hand grip, TUG |
| Li, C. 2021 | **6** | control group habitual diet 6m | 30 | whey protein group | 10g powder(Carbohydrate 0.61g, Fat 0.60g, Protein 7.98g, Amino acids)+100ml water twice/day 6m | 31 | soy protein group | 10g powder(Carbohydrate 0.10g, Fat 0.32g, Protein 8.80g, Amino acids)+100ml water twice/day 6m | 31 | whey-soy blended protein group | 10g poewder(Carbohydrate 0.36g, Fat 0.46g, Protein 8.39g, Amino acids)+100ml water twice/day 6m |  | LBM, hand grip, chair standing, gait speed |
| Li, Z. 2021 | 1,9,10 | control group Healthy education once/2w 3m | 59 | Exercise+Nutrition | Whey protein powder (10 g) 3 times daily  EPA (300 mg), DHA (200 mg), and vitamin D3 (250 IU)  2 pills per time and 2 times daily 5-min warm-up exercise, 20-min strength training, and 5-min slow walking  3 times/w 12w | 59 | Exercise | 5-min warm-up exercise, 20-min strength training, and 5-min slow walking  3 times/w 12w | 62 | Nutrition | Whey protein powder (10 g) 3 times daily  EPA (300 mg), DHA (200 mg), and vitamin D3 (250 IU)  2 pills per time and 2 times daily 12w | 61 | LBM, hand grip |
| Liao, CD. 2018 | 1 | control group | 23 | Elastic resistance exercise | a 10-min warm-up, 40-min period of elastic resistance exercises, and 5-min cool-down period 3 times/w 12w | 33 |  |  |  |  |  |  | LBM, TSM, hand grip, TUG, gait speed |
| Lin, CC. 2021 | 9 | ordinary protein-rich diet | 28 | vitamin D- and leucine-enriched whey protein supplement | a sachet containing supplements included 88 kcal, 12.8 g of protein (including 8.5 g of whey protein concentrate), 1.2 g leucine, 7.3 g carbohydrates, 0.8 g fat and 120 IU vitamin D+200ml water everyday 12w | 57 |  |  |  |  |  |  | body weight, BMI, LBM, gait speed |
| Moghadam, BH. 2020 | 4 | control | 10 | Concurrent Training( a combination of resistance training (RT) and endurance training (ET)) | RT began with 2 sets of 16 to 18 repetitions at 40% of 1RM in the first week and progressed to 3 sets of 8 to 10 repetitions at 75% 1RM in the eighth week ET began at an intensity of 55% of maximum heart rate for 15 minutes in the first week and progressed to 70% of maximum heart rate for 30 minutes in the last week  3times/w 8w | 20 |  |  |  |  |  |  | body weight, BMI |
| Nasimi, N. 2021 | 9 | plain yogurt everyday 12w | 33 | intervention group | 300 g, HMB-, vitamin D-, and vitamin C-fortified yogurt (3 g HMB, 1000 IU vitamin D, and 500 mg vitamin C)  everyday 12 weeks | 33 |  |  |  |  |  |  | BMI, LBM, TSM, hand grip, gait speed |
| Ning, W. 2017 | 5 | no vibration | 20 | whole body vibration training | medium-frequency (MG: 40Hz x 360s), 12w | 20 |  |  |  |  |  |  | chair standing, gait speed |
| Rondanelli, M. 2022 | 8 | placebo | 30 | Novel Food Composed | omega-3 fatty acids (500 mg), leucine (2.5 g), and probiotic Lactobacillus paracasei PS23 (LPPS23) | 30 |  |  |  |  |  |  | body weight, BMI, LBM, hand grip |
| Rondanelli, M. 2018 | 6,8,14 | placebo | 44 | Melatonin | Melatonin 30 min before going to sleep 1 mg/daily | 42 | Essential amino acids | Essential amino acids  every morning during breakfast 4 g/daily 4w | 40 | Melatonin+Essential amino acids | Melatonin 30 min before going to sleep 1 mg/daily Essential amino acids  every morning during breakfast 4 g/daily 4w | 33 | hand grip |
| Sammarco, R. 2017 | 6 | Low-calorie diet plus placebo | 9 | Low-calorie high-protein diet | energy = basal metabolic rate (REE) − 10% according to calorimetry protein intake: 1.2–1.4 g/kg body weight reference/day with 15 g of protein of high biological value for each main meal essential amino acids < branched-chain amino acids < leucine equal to 15 g/day by administration of supplement; carbohydrate: 60–65% of kcal complex fat: to satisfy the required amount of energy; 30% saturated report non-protein kcal/g nitrogen = 100/1 sodium: less than 5 g/day in hypertensive subjects. | 9 |  |  |  |  |  |  | body weight, hand grip |
| Sen, EI. 2021 | 4 | educational program | 44 | home-based training program | posture and stretching exercises, strengthening exercises, balancing training, and a walking regimen 3 days/w 3m | 46 |  |  |  |  |  |  | TUG |
| Seo, MW. 2021 | 1 | control group | 10 | resistance training | five minutes of warm-up, fifty minutes of the resistance exercise, and five minutes of cool-down. 3 times/w 16w | 12 |  |  |  |  |  |  | body weight, BMI, lean body mass, hand grip, gait speed |
| Shahar, S. 2013 | 4,6,10 | control group | 16 | exercise group (ExG) | 10 minutes of general warm-up/aerobic exercises, seven simple balance exercises, 30 minutes of resistance exercises 10 minutes of relaxation exercises twice/w 12w | 19 | protein supplementation group (PrG) | soy protein drin(23% protein, 0.8% fat, and 0.3% carbohydrates 1.5 g/ kg/day  12w | 15 | exercise and protein supplementation group | 10 minutes of general warm-up/aerobic exercises, seven simple balance exercises, 30 minutes of resistance exercises 10 minutes of relaxation exercises twice/w soy protein drin(23% protein, 0.8% fat, and 0.3% carbohydrates 1.5 g/ kg/day  12w | 15 | body weight, BMI, TSM, hand grip |
| Soares Mendes Damasceno, G. 2019 | 13 | control group | 4 | Acupuncture Treatment | 3 times/w | 11 |  |  |  |  |  |  | hand grip |
| Tamura, Y. 2023 | 6 | placebo | 20 | 5-Aminolevulinic Acid Combined with Iron | 100 mg ALA phosphate and 29 mg SFC food intake group | 18 |  |  |  |  |  |  | body weight, BMI, hand grip, chair standing |
| Tsekoura, M. 2018 | 4 | education | 18 | group exercise | 5–10 min warm-up, 20–30-min of strengthening exercises, 20 min of balance and gait training exercises, followed by a 5–10 min cool-down twice/w 12w | 18 |  |  |  |  |  |  | BMI, hand grip, chair standing, gait speed, TUG |
| Vasconcelos, KS. 2016 | 4 | non | 14 | progressive resistance exercise | consisted of a 5-minute walk for warm-up followed by stretching exercises and finally resistance exercises twice/w 10w | 14 |  |  |  |  |  |  | gait speed |
| Vezzoli, A. 2019 | 1 | non | 15 | Moderate Intensity Resistive Training | a 6–8 min aerobic warm-up, followed by 3 series of 14–16 repetitions of chest press, horizontal leg-press, vertical row, and shoulder exercises with free weights (lateral raise) exercises at 60% 1RM 3 times/w 12w | 20 |  |  |  |  |  |  | BMI, TSM, hand grip |
| Wang, R. 2020 | 4 | non | 59 | Mixed Exercise Program | a light 5-minute warm-up, a 20-minute balance exercise, a 5-minute rest, a 20-minute resistance exercise, another 5-minute rest, and a 20-minute aerobic exercise every workday/w 2w | 62 |  |  |  |  |  |  | hand grip, gait speed, TUG |
| Wang, Z. 2022 | 4,8,10 | non | 54 | comprehensive (nutrition plus exercise) groups |  | 60 | nutrition groups |  | 58 | exercise groups |  | 62 | BMI, TSM |
| Wei, M. 2022 | 4 | control group | 30 | Yi Jin Jing and resistance training group | 20 min warm-up,30 min of Yi Jin Jing exercise and 30 min of resistance training | 30 | resistance training group | 60 min of resistance training | 30 |  |  |  | hand grip |
| Xiao, Y. 2023 | 1 | control group | 30 | resistance training | 5-10 min of warm-up exercises, lower limb elastic belt training and squat stand, upper limb elastic belt training, abdominal crunches 6 times/w 4w | 30 |  |  |  |  |  |  | TSM, hand grip |
| Yamada, M. 2019 | 1,9,10 | control group | 28 | combined resistance exercise and nutritional supplementation group | 5 min of warm-up activity, 20 min of the resistance exercise program and 5 min of cool-down activities twice/w Protein and vitamin D supplements (100 kcal; 10.0 g of whey protein, 20 μg [800 IU] of vitamin D and other nutrients) everyday 12w | 28 | the exercise group | 5 min of warm-up activity, 20 min of the resistance exercise program and 5 min of cool-down activities twice/w 12w | 28 | the nutritional supplementation group | Protein and vitamin D supplements (100 kcal; 10.0 g of whey protein, 20 μg [800 IU] of vitamin D and other nutrients) everyday 12w | 28 | LBM, hand grip, chair standing |
| Yin, YH. 2023 | 8 | control group | 30 | Dietary behaviour change intervention | a moderate hypocaloric diet with adequate daily protein intake | 30 |  |  |  |  |  |  | body weight, BMI, hand grip, gait speed |
| Yuenyongchaiwat, K. 2022 | 4 | control group | 30 | pedometer-based walking program plus a resistance exercise program | walking ≥7500 steps daily 5 days/week a resistance exercise use elastic TheraBand twice/w 12w | 30 |  |  |  |  |  |  | hand grip, gait speed |
| Zhu, Y. 2019 | 3,5 | daily lifestyles | 27 | Tai Chi | a 10-min warm-up, 20-min practice, and 10-min relaxation 5 times/w 8w | 24 | whole-body vibration exercise | a 10-min warm-up, 20-min vibration exercise (5 groups/time, and 3 min/group, with 1-min resting between groups), and 10-min relaxation 5 times/w 8w | 28 |  |  |  | TSM, hand grip |

Note: Intervention category (1=resistance (low-moderate load),2=resistance (high load),3=aerobic exercise,4=combined exercise,5=whole-body vibration exercise,6=protein,8=other nutrition,9=protein plus vitamin,10=exercise plus nutrition,13=acupuncture,14=mixed nutrition)

## S3.3 The number of each intervention category

| Intervention category | number |
| --- | --- |
| Exercise prescription |  |
| resistance exercise (low-moderate load) | 10 |
| resistance exercise (high load) | 2 |
| aerobic exercise | 2 |
| combined exercise | 16 |
| whole-body vibration exercise | 3 |
| Nutrition prescription |  |
| protein | 9 |
| other nutrition | 6 |
| protein plus vitamin | 7 |
| mixed nutrition | 10 |
| Comprehensive prescription |  |
| exercise plus nutrition | 1 |
| Other prescription |  |
| acupuncture | 1 |

# Appendix S4: Risk of bias and Quality evaluation of included studies

## S4.1 Quality evaluation of included studies based on Corchane Handbook

| **Author** | **Sequence generation** | **Allocation Concealment** | **Blinding participants and personnel** | **Blinding ofoutcome data** | **Incomplete outcome data** | **Selective outcome reporting** | **Other sourses of bias** |
| --- | --- | --- | --- | --- | --- | --- | --- |
|  |  |  |  |  |  |  |  |
| **Alemán-Mateo H. 2012** | Unclear | Unclear | High | Unclear | Low | Low | Unclear |
| **Bauer, JM. 2015** | Low | Low | Low | Low | Low | Low | Unclear |
| **Bernabei, R. 2022** | Low | Low | Unclear | Low | Low | Low | Unclear |
| **Björkman, MP. 2020** | Low | Low | High | Low | Low | Low | Unclear |
| **Bo, CY. 2019** | Low | Unclear | Low | Unclear | Low | Unclear | Unclear |
| **Chen, BY. 2023** | Low | High | High | High | Low | Low | Unclear |
| **Chen, HT. 2017** | Unclear | Unclear | High | High | Low | Low | Unclear |
| **Chen, HT. 2018** | Unclear | Unclear | High | High | Low | Low | Unclear |
| **Cramer, J. 2015** | Unclear | Unclear | High | High | Low | Low | Low |
| **Ferhi, H. 2023** | Unclear | Low | High | High | Low | Low | Unclear |
| **Flor-Rufino, C. 2023** | Low | High | Low | High | Low | Low | Unclear |
| **Huang, SW. 2017** | Low | Low | Low | High | Low | Low | Unclear |
| **Jung, WS. 2019** | Unclear | Unclear | High | High | Low | Low | Unclear |
| **Kemmler, W. 2018** | Low | Low | Low | High | Low | Low | Unclear |
| **Kemmler, W. 2020** | Low | Low | Low | High | Low | Low | Unclear |
| **Kemmler, W. 2016** | Low | Low | Low | High | Low | Low | Unclear |
| **Kim, H. 2016** | Low | Unclear | High | High | Low | Low | Unclear |
| **Kim, H. 2013** | Low | Low | Low | Low | Low | Low | Unclear |
| **Kim, H. 2012** | Low | Low | Low | Low | Low | Low | Unclear |
| **Kwon, IS. 2021** | Unclear | Unclear | High | High | Low | Low | Unclear |
| **Lee, YH. 2021** | Low | Low | Low | Low | Low | Low | Unclear |
| **Li, C. 2021** | Low | Low | Low | Unclear | Low | Low | Unclear |
| **Li, Z. 2021** | Unclear | Unclear | High | Unclear | Low | Low | Low |
| **Liao, CD. 2018** | Low | High | Low | Unclear | Low | Low | Unclear |
| **Lin, CC. 2021** | Unclear | Unclear | High | Unclear | Low | Low | Low |
| **Moghadam, BH. 2020** | Unclear | Unclear | High | Unclear | Low | Low | Unclear |
| **Nasimi, N. 2021** | Unclear | Low | Low | Low | Low | Low | Unclear |
| **Ning, W. 2017** | Low | Unclear | Unclear | Unclear | Low | Low | Unclear |
| **Rondanelli, M. 2022** | Low | Low | Unclear | Unclear | Low | Low | Unclear |
| **Rondanelli, M. 2018** | Low | Unclear | Unclear | Unclear | Low | Low | Low |
| **Sammarco, R. 2017** | Unclear | Unclear | High | High | Low | Low | Unclear |
| **Sen, EI. 2021** | Unclear | Unclear | High | High | Low | Low | Unclear |
| **Seo, MW. 2021** | Low | Unclear | High | High | Low | Low | Unclear |
| **Shahar, S. 2013** | Unclear | Unclear | High | High | Low | Low | Low |
| **Soares Mendes Damasceno, G. 2019** | Low | Unclear | High | High | Low | Low | Low |
| **Tamura, Y. 2023** | Low | Low | Unclear | Unclear | Low | Low | Unclear |
| **Tsekoura, M. 2018** | Low | Low | High | High | Low | Low | Low |
| **Vasconcelos, KS. 2016** | Unclear | Unclear | High | High | Low | Low | Unclear |
| **Vezzoli, A. 2019** | Unclear | Unclear | High | High | Low | Low | Unclear |
| **Wang, R. 2020** | High | Unclear | High | High | Low | Low | Unclear |
| **Wang, Z. 2022** | Unclear | Unclear | High | High | Low | Low | Low |
| **Wei, M. 2022** | Unclear | Unclear | High | High | Low | Low | Unclear |
| **Xiao, Y. 2023** | Low | Low | Unclear | Low | Low | Low | Unclear |
| **Yamada, M. 2019** | Low | Unclear | High | Low | Low | Low | Unclear |
| **Yin, YH. 2023** | Low | Low | High | Low | Low | Low | Unclear |
| **Yuenyongchaiwat, K. 2022** | Unclear | Unclear | High | Low | Low | Low | Unclear |
| **Zhu, Y. 2019** | Unclear | Unclear | High | High | Low | Low | Unclear |

## S4.2 Reliability of network meta-analysis primary results based on CINeMA

**Table1． Reliability of skeletal muscle mass based on CINeMA**

| Comparison | Number | Within-study bias | Reporting bias | Indirectness | Imprecision | Heterogeneity | Incoherence | Confidence rating |
| --- | --- | --- | --- | --- | --- | --- | --- | --- |
| AE:placebo | 2 | Major concerns | Low risk | No concerns | Major concerns | No concerns | No concerns | High |
| CE:placebo | 6 | Some concerns | Low risk | No concerns | Major concerns | No concerns | No concerns | Moderate |
| EplusN:placebo | 4 | Some concerns | Low risk | No concerns | No concerns | No concerns | No concerns | Very low |
| ON:placebo | 3 | Major concerns | Low risk | No concerns | No concerns | No concerns | No concerns | Low |
| placebo:PT | 3 | No concerns | Low risk | No concerns | Major concerns | No concerns | No concerns | Low |
| placebo:PplusV | 2 | Major concerns | Low risk | No concerns | Major concerns | No concerns | No concerns | High |
| placebo:RHL | 1 | Major concerns | Low risk | No concerns | Major concerns | No concerns | No concerns | High |
| placebo:RLML | 7 | Some concerns | Low risk | No concerns | No concerns | No concerns | No concerns | Very low |
| placebo:WBVE | 1 | Major concerns | Low risk | No concerns | Major concerns | No concerns | No concerns | High |

**Table2． Reliability of lean body mass based on CINeMA**

| Comparison | Number | Within-study bias | Reporting bias | Indirectness | Imprecision | Heterogeneity | Incoherence | Confidence rating |
| --- | --- | --- | --- | --- | --- | --- | --- | --- |
| AE:placebo | 1 | Major concerns | Low risk | No concerns | Major concerns | No concerns | No concerns | High |
| CE:placebo | 7 | Some concerns | Low risk | No concerns | No concerns | Major concerns | Major concerns | High |
| EplusN:placebo | 7 | Some concerns | Low risk | No concerns | No concerns | Major concerns | No concerns | Moderate |
| ON:placebo | 3 | Major concerns | Low risk | No concerns | Major concerns | No concerns | Major concerns | High |
| placebo:PT | 4 | No concerns | Low risk | No concerns | Major concerns | No concerns | No concerns | Low |
| placebo:PplusV | 5 | Some concerns | Low risk | No concerns | No concerns | Major concerns | No concerns | Moderate |
| placebo:RHL | 1 | Some concerns | Low risk | No concerns | Major concerns | No concerns | No concerns | Moderate |
| placebo:RLML | 7 | Some concerns | Low risk | No concerns | No concerns | Major concerns | No concerns | Moderate |

# Appendix S5: Consistency assessment

## S5.1 The PSRF value of each outcome.

| skeletal muscle mass | Multivariate psrf: 1 | | lean body mass | Multivariate psrf: 1 | | handgrip strength | Multivariate psrf: 1 | |
| --- | --- | --- | --- | --- | --- | --- | --- | --- |
|  | Point est. | Upper C.I. |  | Point est. | Upper C.I. |  | Point est. | Upper C.I. |
| placebo vs RLML | 1 | 1 | placebo vs RLML | 1 | 1 | placebo vs RLML | 1 | 1 |
| placebo vs RHL | 1 | 1 | placebo vs RHL | 1 | 1 | placebo vs RHL | 1 | 1 |
| placebo vs AE | 1 | 1 | placebo vs AE | 1 | 1 | placebo vs AE | 1 | 1 |
| placebo vs CE | 1 | 1 | placebo vs CE | 1 | 1 | placebo vs CE | 1 | 1 |
| placebo vs WBVE | 1 | 1 | placebo vs PT | 1 | 1 | placebo vs WBVE | 1 | 1 |
| placebo vs PT | 1 | 1 | placebo vs ON | 1 | 1 | placebo vs PT | 1 | 1 |
| placebo vs ON | 1 | 1 | placebo vs PplusV | 1 | 1 | placebo vs ON | 1 | 1 |
| placebo vs PplusV | 1 | 1 | placebo vs EplusN | 1 | 1 | placebo vs PplusV | 1 | 1 |
| placebo vs EplusN | 1 | 1 |  |  |  | placebo vs EplusN | 1 | 1 |
|  |  |  |  |  |  | placebo vs ACU | 1 | 1 |
|  |  |  |  |  |  | placebo vs MN | 1 | 1 |
| Gait speed | Multivariate psrf: 1 | | **TUG** | Multivariate psrf: 1 | | **chair standing** | Multivariate psrf: 1 | |
|  | Point est. | Upper C.I. |  | Point est. | Upper C.I. |  | Point est. | Upper C.I. |
| placebo vs RLML | 1 | 1 | placebo vs RLML | 1 | 1 | placebo vs RLML | 1 | 1 |
| placebo vs RHL | 1 | 1 | placebo vs CE | 1 | 1 | placebo vs CE | 1 | 1 |
| placebo vs CE | 1 | 1.02 | placebo vs WBVE | 1 | 1 | placebo vs WBVE | 1 | 1 |
| placebo vs WBVE | 1 | 1 | placebo vs PT | 1 | 1 | placebo vs PT | 1 | 1 |
| placebo vs PT | 1 | 1 | placebo vs ON | 1 | 1 | placebo vs PplusV | 1 | 1 |
| placebo vs ON | 1 | 1 | placebo vs EplusN | 1 | 1 | placebo vs EplusN | 1 | 1 |
| placebo vs PplusV | 1 | 1 |  |  |  |  |  |  |
| placebo vs EplusN | 1 | 1 |  |  |  |  |  |  |
| body weight | Multivariate psrf: 1 | | **BMI** | Multivariate psrf: 1 | | **SPPB** | Multivariate psrf: 1 | |
|  | Point est. | Upper C.I. |  | Point est. | Upper C.I. |  | Point est. | Upper C.I. |
| placebo vs RLML | 1 | 1 | placebo vs RLML | 1 | 1 | placebo vs RLML | 1 | 1 |
| placebo vs RHL | 1 | 1 | placebo vs RHL | 1 | 1 | placebo vs RHL | 1 | 1 |
| placebo vs AE | 1 | 1 | placebo vs AE | 1 | 1 | placebo vs CE | 1 | 1 |
| placebo vs CE | 1 | 1 | placebo vs CE | 1 | 1 | placebo vs PT | 1 | 1 |
| placebo vs PT | 1 | 1 | placebo vs PT | 1 | 1 | placebo vs ON | 1 | 1 |
| placebo vs ON | 1 | 1 | placebo vs ON | 1 | 1 | placebo vs PplusV | 1 | 1 |
| placebo vs PplusV | 1 | 1 | placebo vs PplusV | 1 | 1 |  |  |  |
| placebo vs EplusN | 1 | 1 | placebo vs EplusN | 1 | 1 |  |  |  |
| SF-36 | Multivariate psrf: 1 | |  |  |  |  |  |  |
|  | Point est. | Upper C.I. |  |  |  |  |  |  |
| placebo vs RLML | 1 | 1 |  |  |  |  |  |  |
| placebo vs CE | 1 | 1 |  |  |  |  |  |  |
| placebo vs PT | 1 | 1 |  |  |  |  |  |  |
| placebo vs ON | 1 | 1 |  |  |  |  |  |  |
| placebo vs PplusV | 1 | 1 |  |  |  |  |  |  |

# Appendix S6: Results of SUCRA

|  | placebo | RLML | RHL | AE | CE | WBVE | PT | ON | PplusV | EplusN | ACU | MN |
| --- | --- | --- | --- | --- | --- | --- | --- | --- | --- | --- | --- | --- |
| skeletal muscle mass | 10.8% | 62.6% | 86.0% | 62.7% | 31.5% | 57.0% | 26.2% | 74.5% | 27.4% | 61.4% |  |  |
| lean body mass | 9.5% | 65.1% | 11.3% | 58.7% | 53.3% |  | 46.7% | 72.6% | 55.2% | 77.5% |  |  |
| handgrip strength | 13.5% | 78.6% | 74.6% | 22.1% | 49.2% | 56.7% | 34.9% | 66.7% | 63.1% | 66.1% | 41.2% | 33.4% |
| gait speed | 22.2% | 96.7% | 15.6% |  | 85.1% | 73.4% | 65.3% | 1.1% | 39.3% | 51.2% |  |  |
| time up and go test | 6.9% | 63.5% |  |  | 77.0% | 86.5% | 29.2% | 26.6% |  | 60.3% |  |  |
| chair standing | 56.3% | 45.9% | 45.0% | 46.6% | 20.9% |  | 34.0% | 86.6% | 46.7% | 68.1% |  |  |
| body weight | 52.1% | 53.5% | 63.7% | 50.7% | 22.4% |  | 64.8% | 31.7% | 79.9% | 31.3% |  |  |
| body mass index | 43.6% | 54.0% | 54.9% | 53.9% | 79.0% |  | 65.9% | 13.3% | 53.2% | 32.0% |  |  |

# Appendix S7: Results of Meta-regression

## S7.1 skeletal muscle mass

Figure1. meta regression based on age


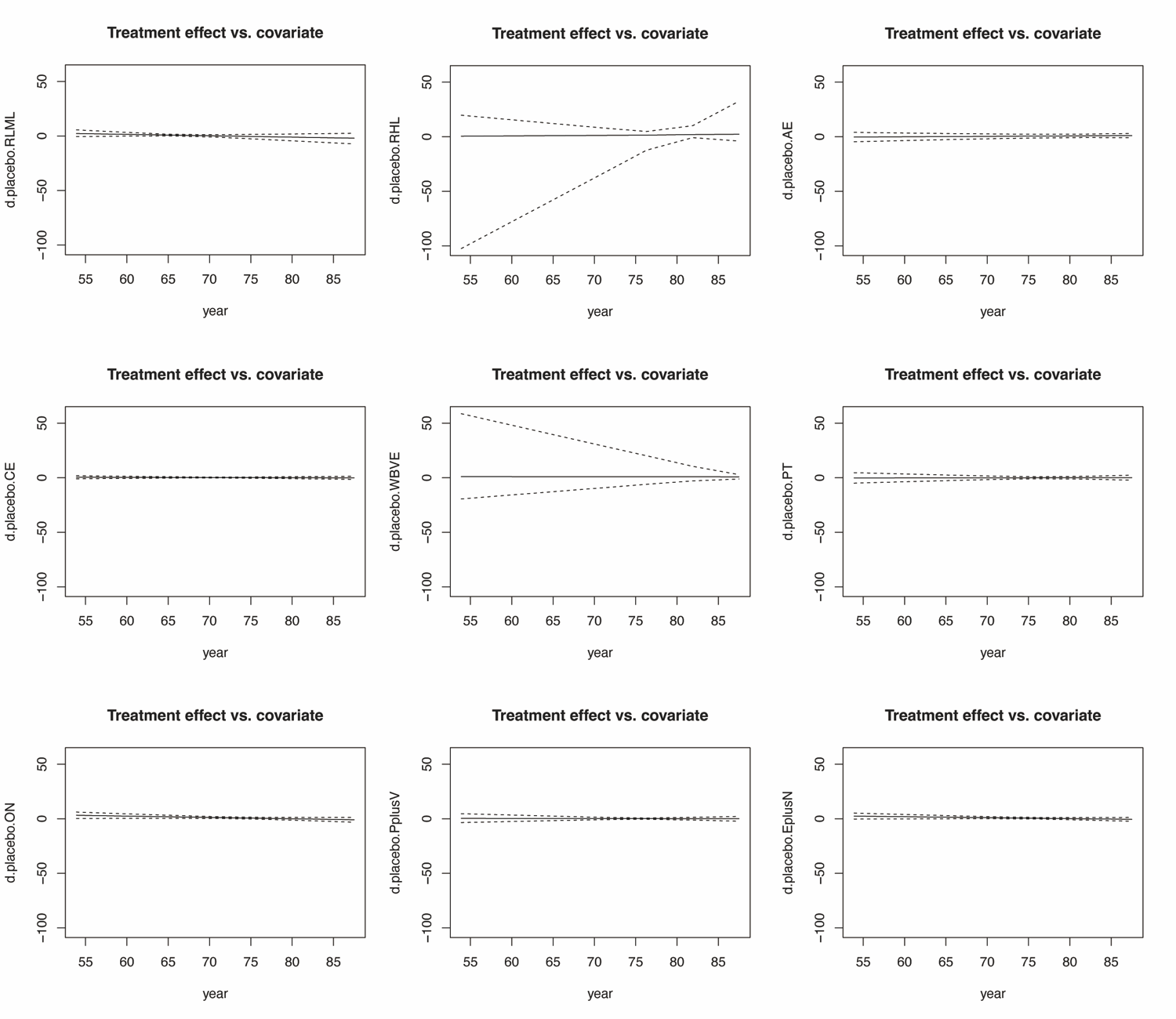


Figure2. meta regression based on BMI


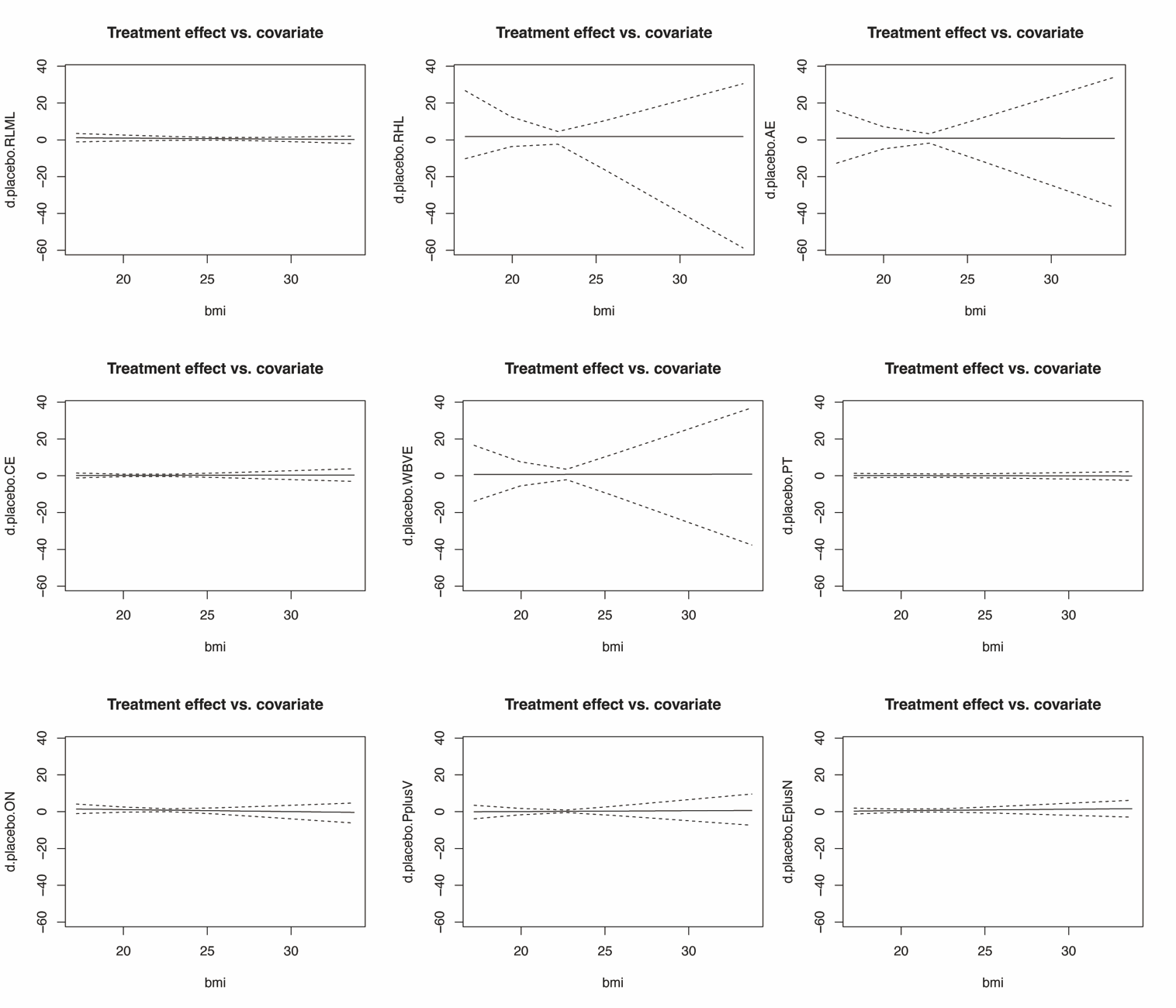


Figure3. meta regression based on duration


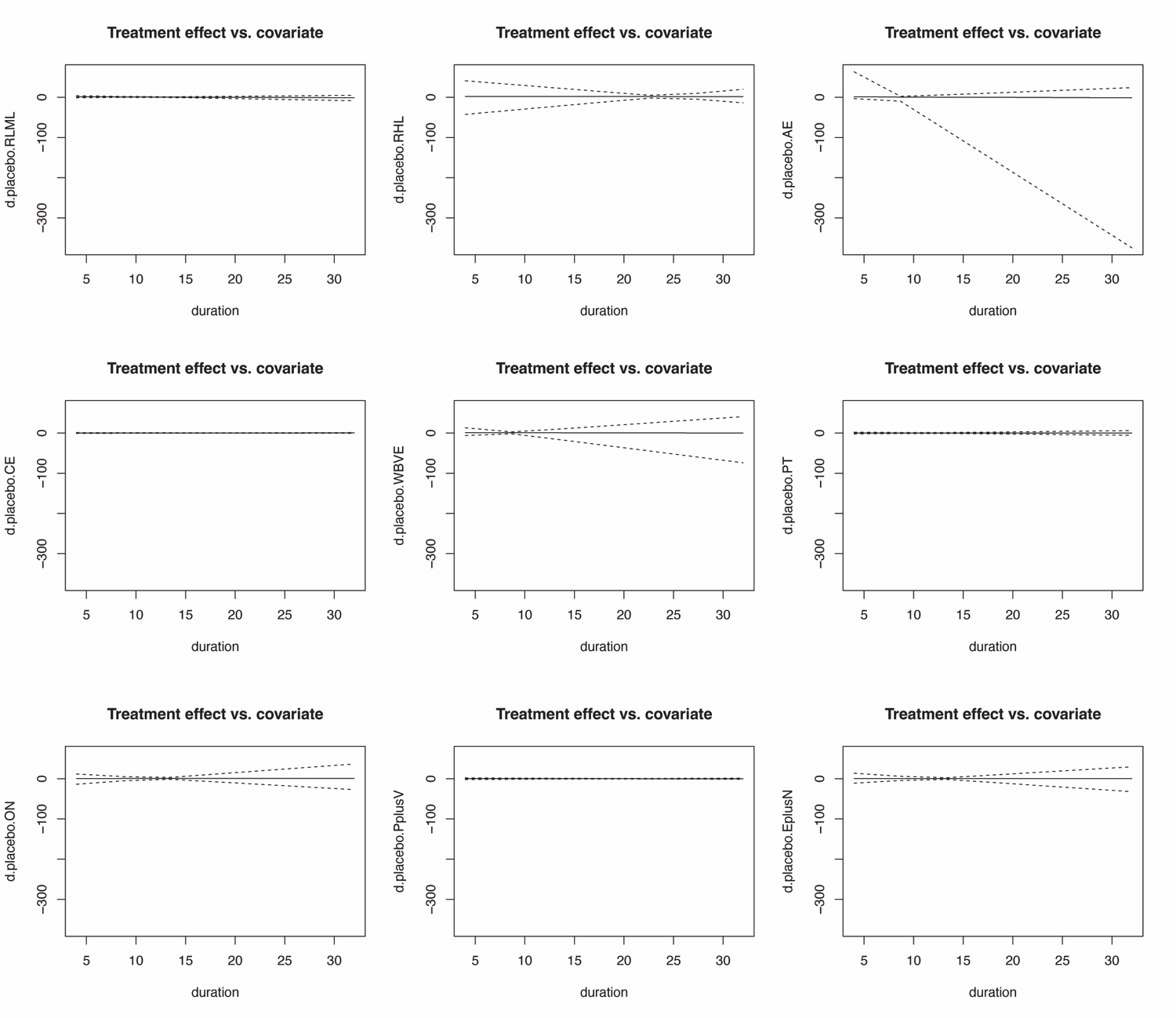


Figure4. meta regression based on diagnosis criterior


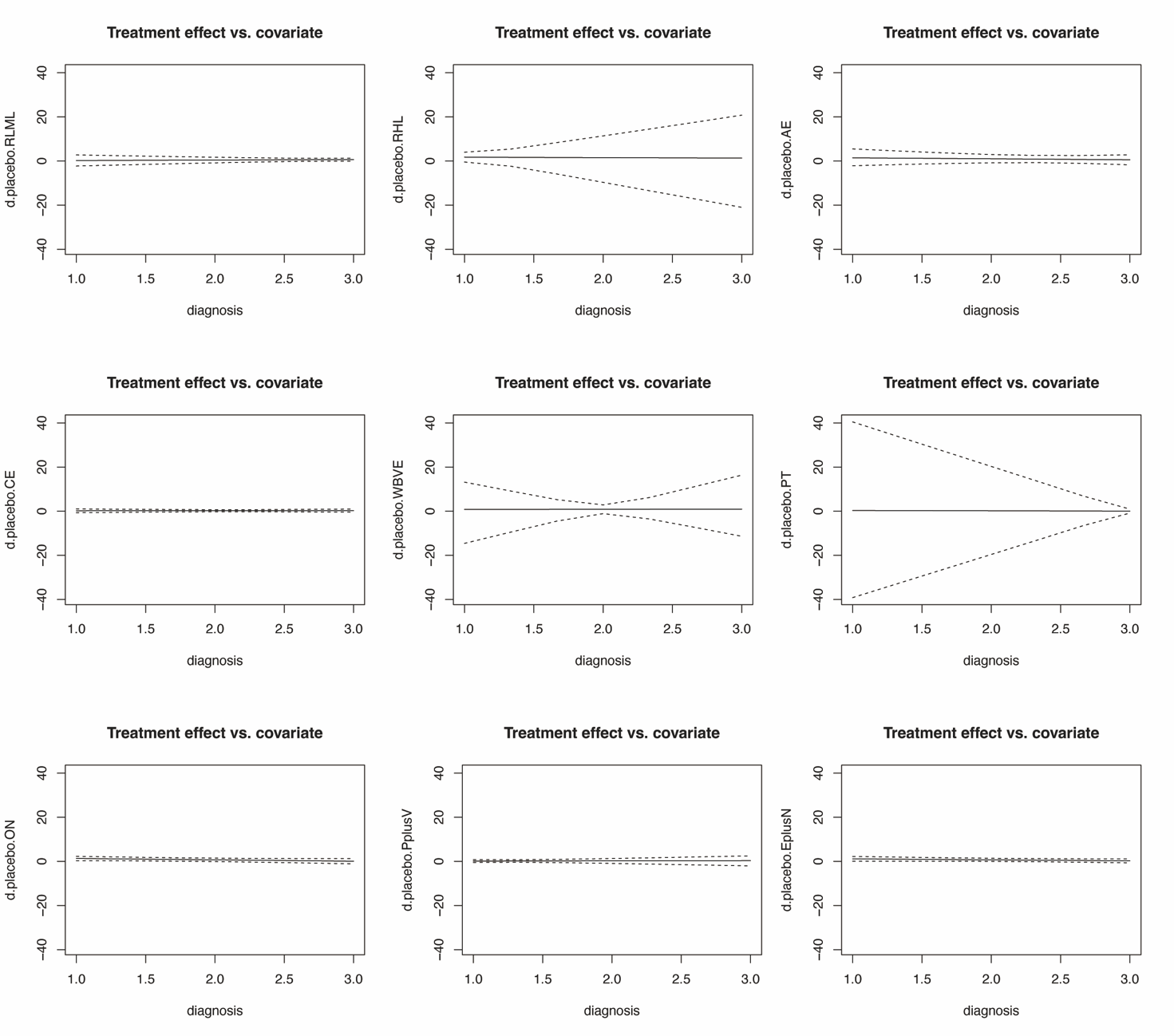


Figure5. meta regression based on participants number


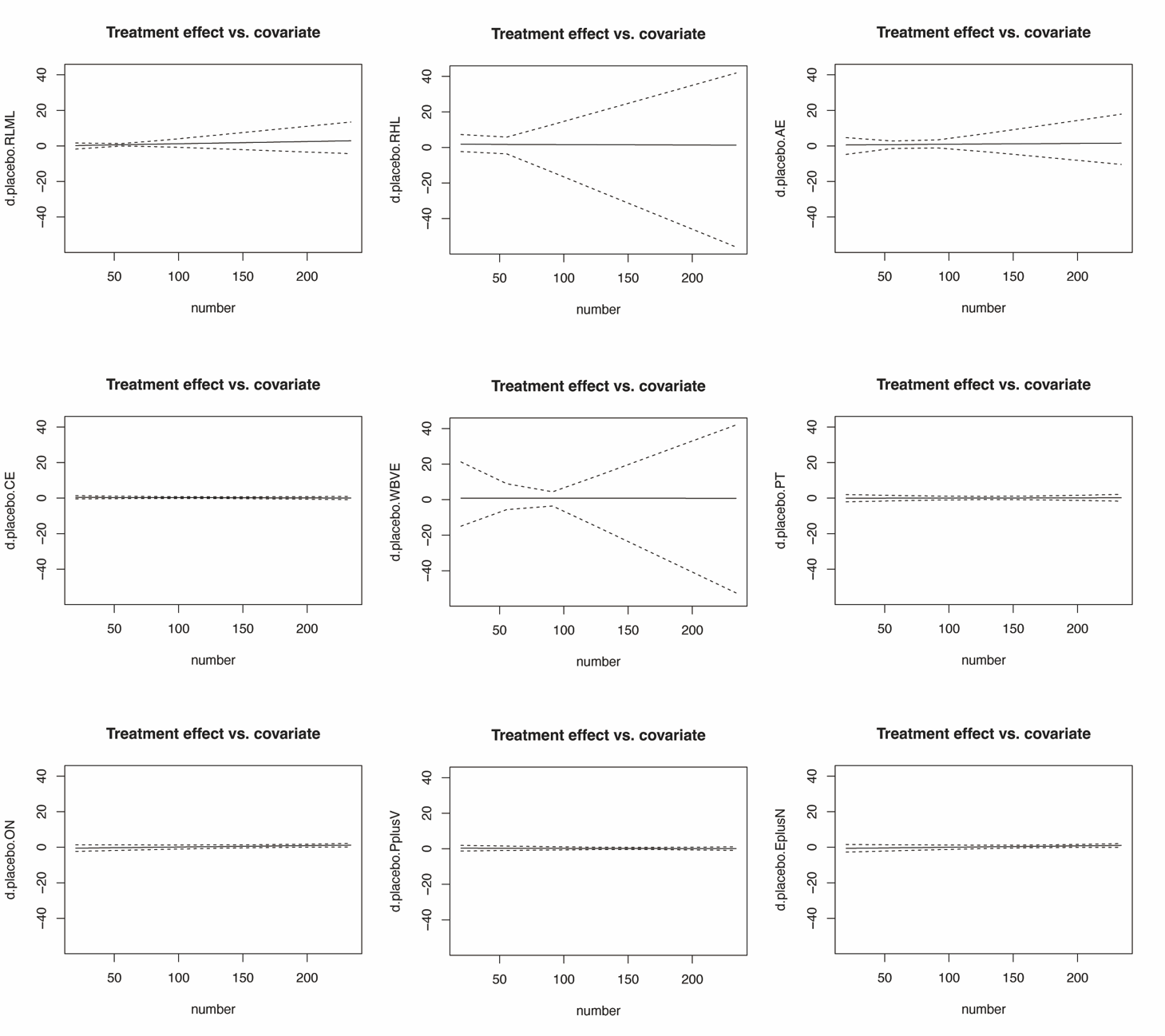


## S7.2 Lean body mass

Figure1. meta regression based on age


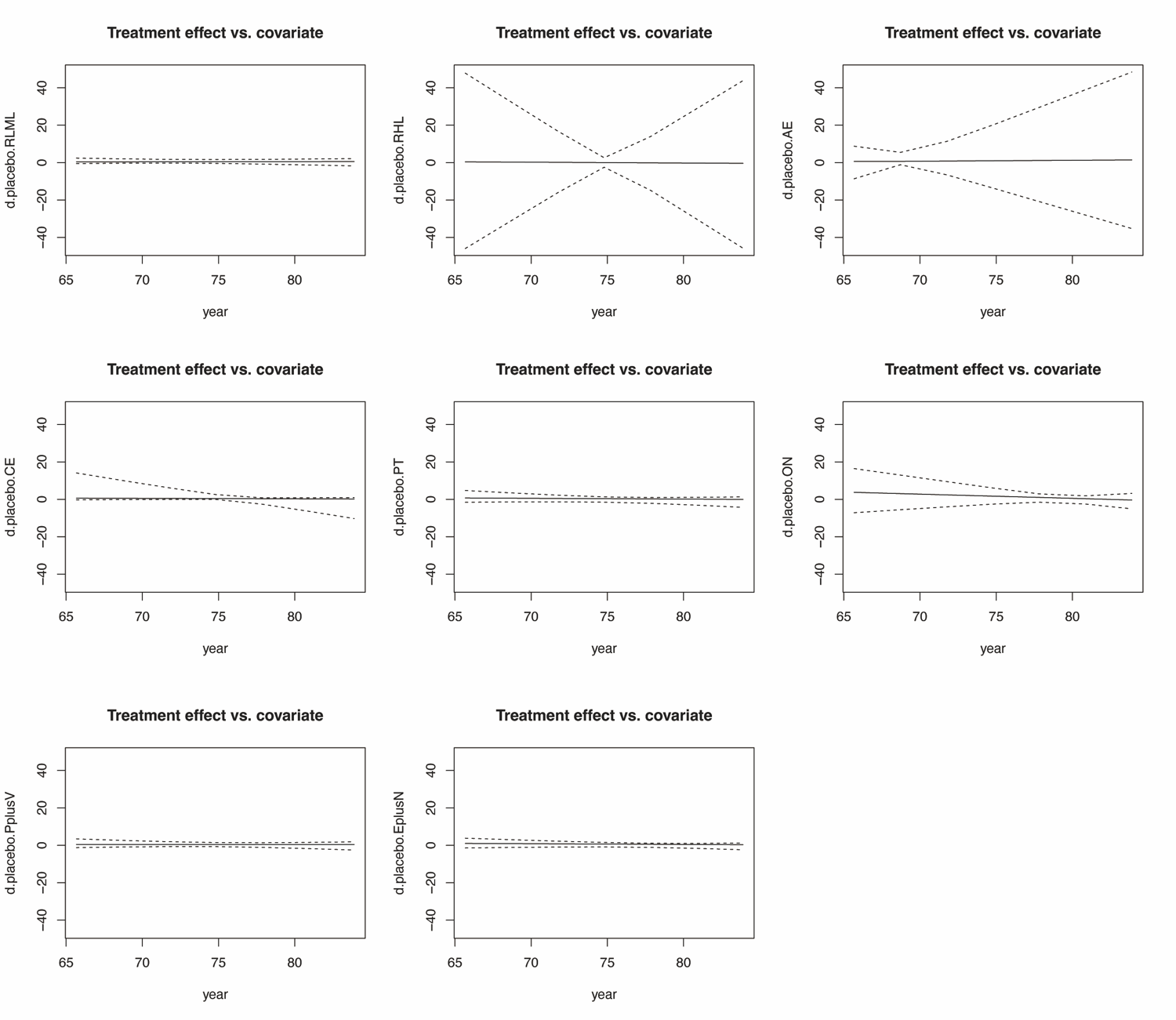


Figure2. meta regression based on BMI


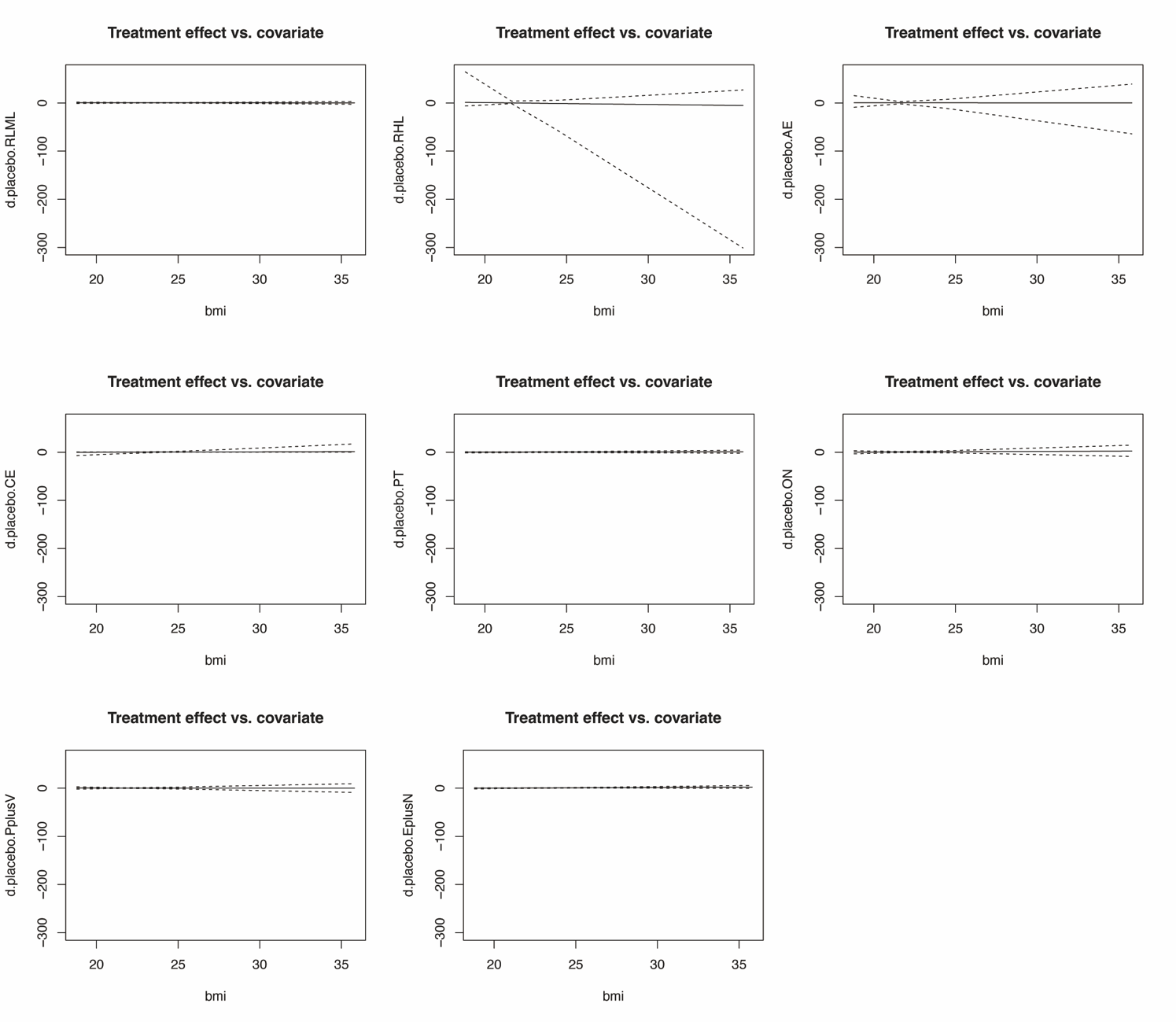


Figure3. meta regression based on diagnosis criterior


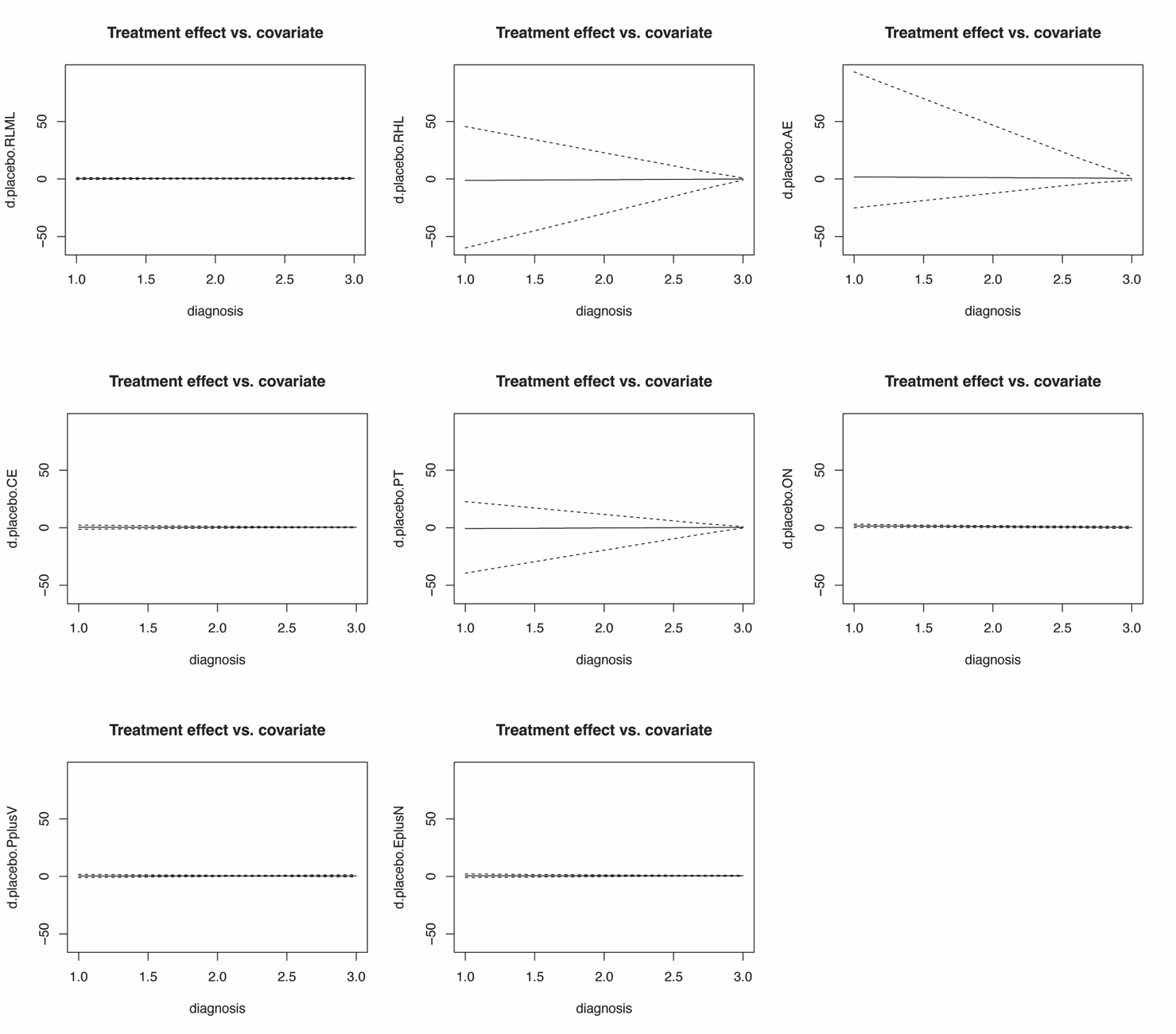


Figure4. meta regression based on duration


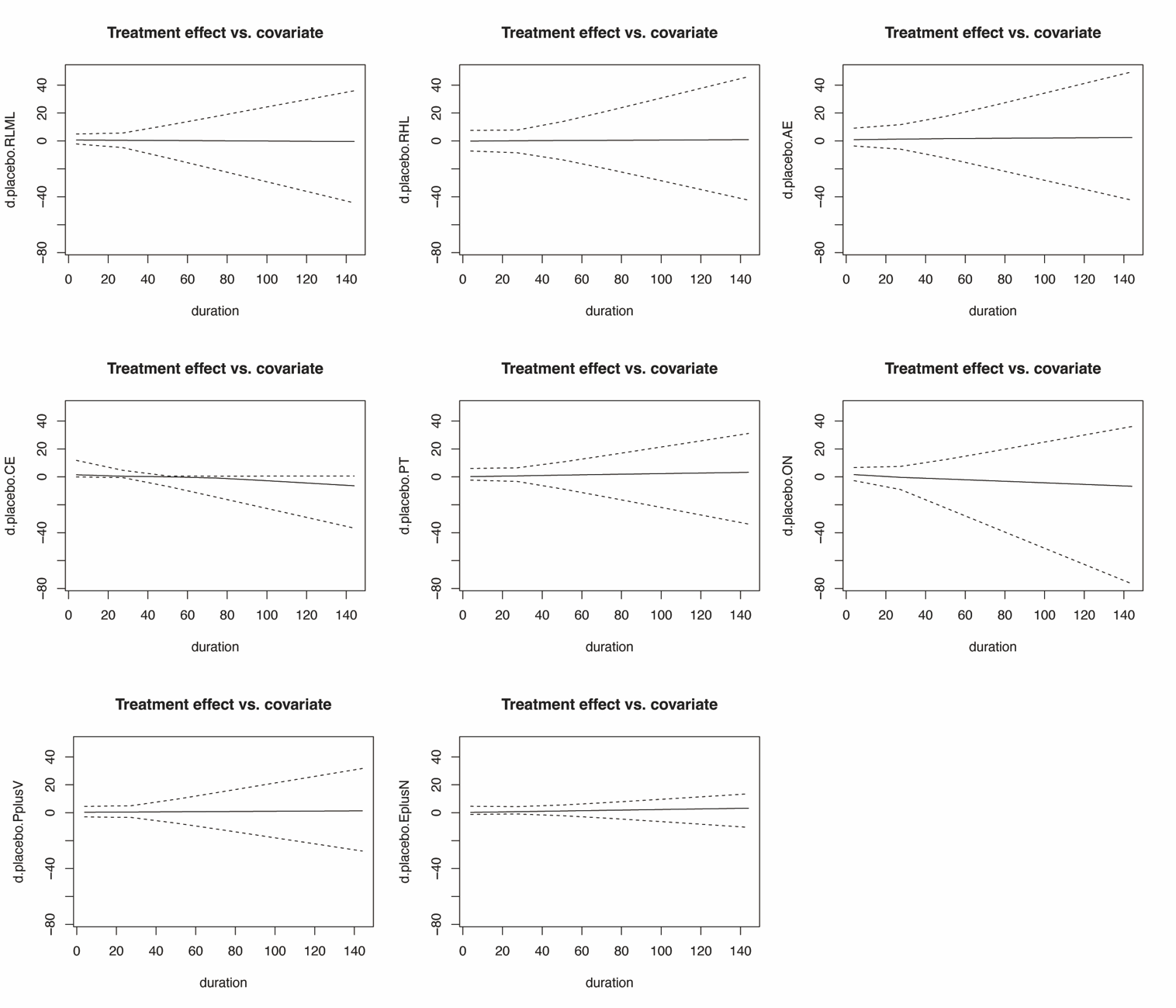


Figure5. meta regression based on participants number


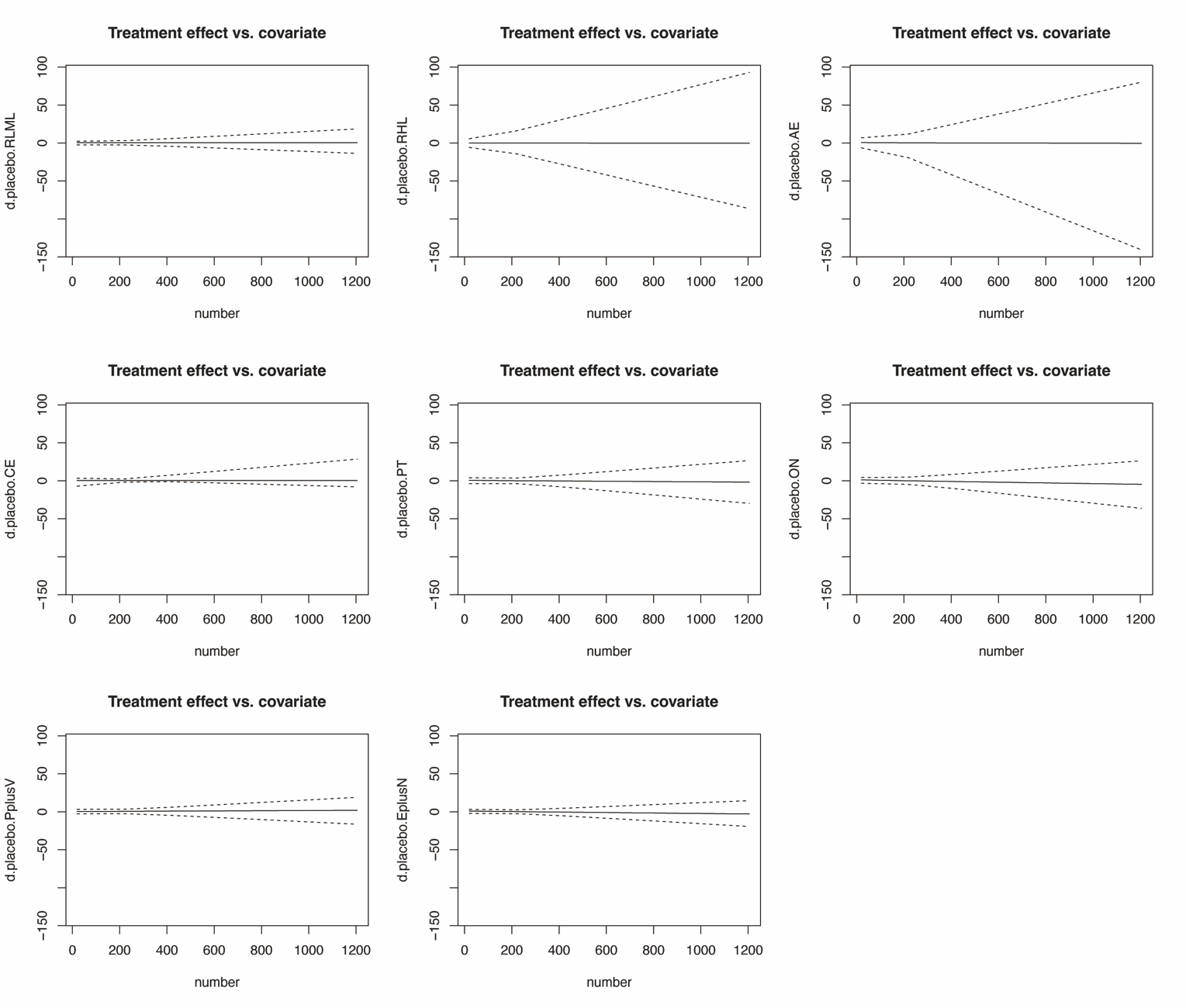


## S7.3 The funnel plot of skeletal muscle mass and lean body mass


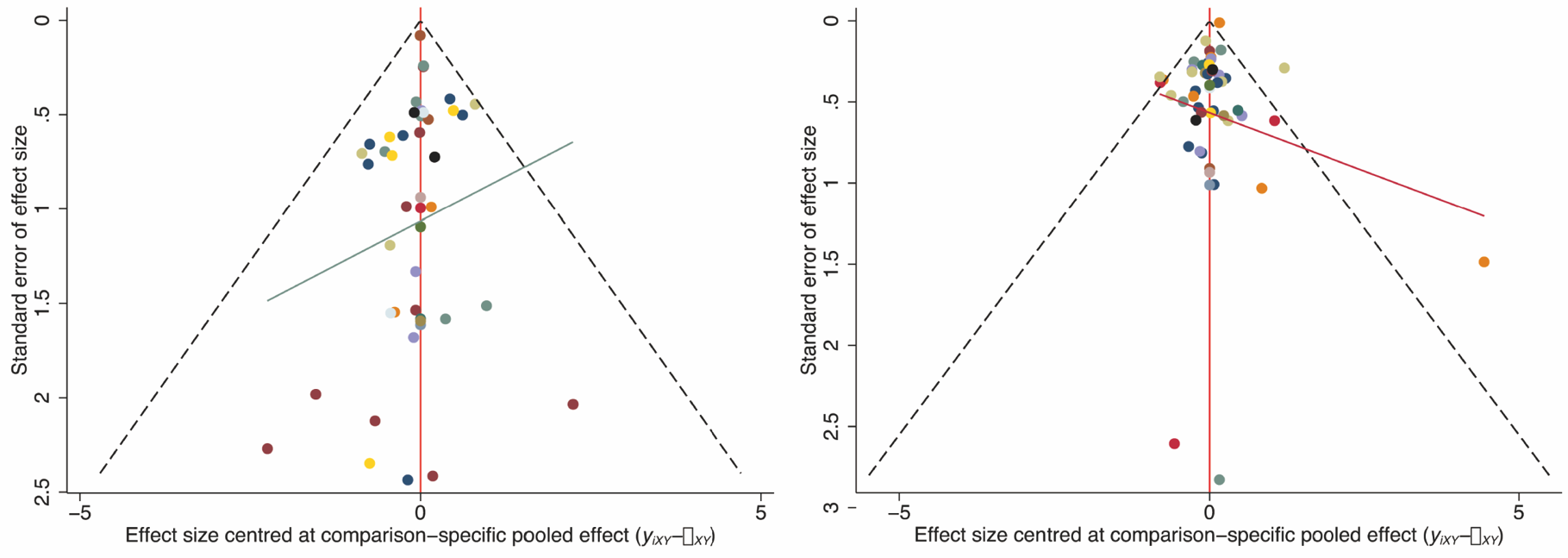


Note: Left one is skeletal muscle mass, right one is lean body mass

Reference

1. Alemán-Mateo H, Macías L, Esparza-Romero J, Astiazaran-García H, Blancas AL: **Physiological effects beyond the significant gain in muscle mass in sarcopenic elderly men: evidence from a randomized clinical trial using a protein-rich food**. *Clin Interv Aging* 2012, **7**:225-234.

2. Bauer JM, Verlaan S, Bautmans I, Brandt K, Donini LM, Maggio M, McMurdo ME, Mets T, Seal C, Wijers SL *et al*: **Effects of a vitamin D and leucine-enriched whey protein nutritional supplement on measures of sarcopenia in older adults, the PROVIDE study: a randomized, double-blind, placebo-controlled trial**. *J Am Med Dir Assoc* 2015, **16**(9):740-747.

3. Bernabei R, Landi F, Calvani R, Cesari M, Del Signore S, Anker SD, Bejuit R, Bordes P, Cherubini A, Cruz-Jentoft AJ *et al*: **Multicomponent intervention to prevent mobility disability in frail older adults: randomised controlled trial (SPRINTT project)**. *Bmj* 2022, **377**:e068788.

4. Björkman MP, Suominen MH, Kautiainen H, Jyväkorpi SK, Finne-Soveri HU, Strandberg TE, Pitkälä KH, Tilvis RS: **Effect of Protein Supplementation on Physical Performance in Older People With Sarcopenia-A Randomized Controlled Trial**. *J Am Med Dir Assoc* 2020, **21**(2):226-232.e221.

5. Bo Y, Liu C, Ji Z, Yang R, An Q, Zhang X, You J, Duan D, Sun Y, Zhu Y *et al*: **A high whey protein, vitamin D and E supplement preserves muscle mass, strength, and quality of life in sarcopenic older adults: A double-blind randomized controlled trial**. *Clin Nutr* 2019, **38**(1):159-164.

6. Chen B-Y, Chen Y-Z, Shin S-H, Jie C-Y, Chang Z-L, Ding H, Yang H: **Effect of a moderate-intensity comprehensive exercise program on body composition, muscle strength, and physical performance in elderly females with sarcopenia**. *Heliyon* 2023, **9**(8):e18951.

7. Chen H-T, Chung Y-C, Chen Y-J, Ho S-Y, Wu H-J: **Effects of Different Types of Exercise on Body Composition, Muscle Strength, and IGF-1 in the Elderly with Sarcopenic Obesity**. *Journal of the American Geriatrics Society* 2017, **65**(4):827-832.

8. Chen HT, Wu HJ, Chen YJ, Ho SY, Chung YC: **Effects of 8-week kettlebell training on body composition, muscle strength, pulmonary function, and chronic low-grade inflammation in elderly women with sarcopenia**. *Exp Gerontol* 2018, **112**:112-118.

9. Cramer J, Cruz-Jentoft AJ, Hickson M, Landi F, Zamboni M, Pereira S, Hustead D, Mustad V: **High-protein oral nutritional supplements improve leg muscle quality in sarcopenic, malnourished adults**. *European Geriatric Medicine* 2015, **6**:S90.

10. Ferhi H, Gaied Chortane S, Durand S, Beaune B, Boyas S, Maktouf W: **Effects of Physical Activity Program on Body Composition, Physical Performance, and Neuromuscular Strategies during Walking in Older Adults with Sarcopenic Obesity: Randomized Controlled Trial**. *Healthcare (Basel)* 2023, **11**(16).

11. Flor-Rufino C, Barrachina-Igual J, Pérez-Ros P, Pablos-Monzó A, Sanz-Requena R, Martínez-Arnau FM: **Fat infiltration and muscle hydration improve after high-intensity resistance training in women with sarcopenia. A randomized clinical trial**. *Maturitas* 2023, **168**:29-36.

12. Huang SW, Ku JW, Lin LF, Liao CD, Chou LC, Liou TH: **Body composition influenced by progressive elastic band resistance exercise of sarcopenic obesity elderly women: a pilot randomized controlled trial**. *Eur J Phys Rehabil Med* 2017, **53**(4):556-563.

13. Jung W-S, Kim Y-Y, Park H-Y: **Circuit Training Improvements in Korean Women with Sarcopenia**. *Perceptual and motor skills* 2019, **126**(5):828-842.

14. Kemmler W, Grimm A, Bebenek M, Kohl M, von Stengel S: **Effects of Combined Whole-Body Electromyostimulation and Protein Supplementation on Local and Overall Muscle/Fat Distribution in Older Men with Sarcopenic Obesity: The Randomized Controlled Franconia Sarcopenic Obesity (FranSO) Study**. *Calcif Tissue Int* 2018, **103**(3):266-277.

15. Kemmler W, Kohl M, Fröhlich M, Jakob F, Engelke K, von Stengel S, Schoene D: **Effects of High-Intensity Resistance Training on Osteopenia and Sarcopenia Parameters in Older Men with Osteosarcopenia-One-Year Results of the Randomized Controlled Franconian Osteopenia and Sarcopenia Trial (FrOST)**. *J Bone Miner Res* 2020, **35**(9):1634-1644.

16. Kemmler W, Teschler M, Weissenfels A, Bebenek M, von Stengel S, Kohl M, Freiberger E, Goisser S, Jakob F, Sieber C *et al*: **Whole-body electromyostimulation to fight sarcopenic obesity in community-dwelling older women at risk. Resultsof the randomized controlled FORMOsA-sarcopenic obesity study**. *Osteoporos Int* 2016, **27**(11):3261-3270.

17. Kim H, Kim M, Kojima N, Fujino K, Hosoi E, Kobayashi H, Somekawa S, Niki Y, Yamashiro Y, Yoshida H: **Exercise and Nutritional Supplementation on Community-Dwelling Elderly Japanese Women With Sarcopenic Obesity: A Randomized Controlled Trial**. *J Am Med Dir Assoc* 2016, **17**(11):1011-1019.

18. Kim H, Suzuki T, Saito K, Yoshida H, Kojima N, Kim M, Sudo M, Yamashiro Y, Tokimitsu I: **Effects of exercise and tea catechins on muscle mass, strength and walking ability in community-dwelling elderly Japanese sarcopenic women: a randomized controlled trial**. *Geriatr Gerontol Int* 2013, **13**(2):458-465.

19. Kim HK, Suzuki T, Saito K, Yoshida H, Kobayashi H, Kato H, Katayama M: **Effects of exercise and amino acid supplementation on body composition and physical function in community-dwelling elderly Japanese sarcopenic women: a randomized controlled trial**. *J Am Geriatr Soc* 2012, **60**(1):16-23.

20. Kwon I-S, Park D-S, Shin H-C, Seok M-G, Oh J-K: **Effects of marine oligomeric polyphenols on body composition and physical ability of elderly individuals with sarcopenia: a pilot study**. *Physical activity and nutrition* 2021, **25**(3):1-7.

21. Lee Y-H, Lee P-H, Lin L-F, Liao C-D, Liou T-H, Huang S-W: **Effects of progressive elastic band resistance exercise for aged osteosarcopenic adiposity women**. *Experimental gerontology* 2021, **147**:111272.

22. Li C, Meng H, Wu S, Fang A, Liao G, Tan X, Chen P, Wang X, Chen S, Zhu H: **Daily Supplementation With Whey, Soy, or Whey-Soy Blended Protein for 6 Months Maintained Lean Muscle Mass and Physical Performance in Older Adults With Low Lean Mass**. *J Acad Nutr Diet* 2021, **121**(6):1035-1048.e1036.

23. Li Z, Cui M, Yu K, Zhang X-W, Li C-W, Nie X-D, Wang F: **Effects of nutrition supplementation and physical exercise on muscle mass, muscle strength and fat mass among sarcopenic elderly: a randomized controlled trial**. *Applied physiology, nutrition, and metabolism = Physiologie appliquee, nutrition et metabolisme* 2021, **46**(5):494-500.

24. Liao C-D, Tsauo J-Y, Huang S-W, Ku J-W, Hsiao D-J, Liou T-H: **Effects of elastic band exercise on lean mass and physical capacity in older women with sarcopenic obesity: A randomized controlled trial**. *Scientific reports* 2018, **8**(1):2317.

25. Lin CC, Shih MH, Chen CD, Yeh SL: **Effects of adequate dietary protein with whey protein, leucine, and vitamin D supplementation on sarcopenia in older adults: An open-label, parallel-group study**. *Clinical Nutrition* 2021, **40**(3):1323-1329.

26. Moghadam BH, Bagheri R, Ashtary-Larky D, Tinsley GM, Eskandari M, Wong A, Kreider RB, Baker JS: **The Effects of Concurrent Training Order on Satellite Cell-Related Markers, Body Composition, Muscular and Cardiorespiratory Fitness in Older Men with Sarcopenia**. *Journal of Nutrition, Health and Aging* 2020, **24**(7):796-804.

27. Nasimi N, Sohrabi Z, Dabbaghmanesh MH, Eskandari MH, Bedeltavana A, Famouri M, Talezadeh P: **A Novel Fortified Dairy Product and Sarcopenia Measures in Sarcopenic Older Adults: A Double-Blind Randomized Controlled Trial**. *J Am Med Dir Assoc* 2021, **22**(4):809-815.

28. Ning W, Pang MYC, Ng SSM, Ng GYF: **Optimal frequency/time combination of whole body vibration training for developing physical performance of people with sarcopenia: a randomized controlled trial**. *Clinical rehabilitation* 2017, **31**(10):1313‐1321.

29. Rondanelli M, Gasparri C, Barrile GC, Battaglia S, Cavioni A, Giusti R, Mansueto F, Moroni A, Nannipieri F, Patelli Z *et al*: **Effectiveness of a Novel Food Composed of Leucine, Omega-3 Fatty Acids and Probiotic Lactobacillus paracasei PS23 for the Treatment of Sarcopenia in Elderly Subjects: a 2-Month Randomized Double-Blind Placebo-Controlled Trial**. *Nutrients* 2022, **14**(21).

30. Rondanelli M, Peroni G, Gasparri C, Infantino V, Nichetti M, Cuzzoni G, Spadaccini D, Perna S: **Is a Combination of Melatonin and Amino Acids Useful to Sarcopenic Elderly Patients? A Randomized Trial**. *Geriatrics (Basel)* 2018, **4**(1).

31. Sammarco R, Marra M, Di Guglielmo ML, Naccarato M, Contaldo F, Poggiogalle E, Donini LM, Pasanisi F: **Evaluation of Hypocaloric Diet With Protein Supplementation in Middle-Aged Sarcopenic Obese Women: A Pilot Study**. *Obes Facts* 2017, **10**(3):160-167.

32. Sen EI, Eyigor S, Dikici Yagli M, Ozcete ZA, Aydin T, Kesiktas FN, Aydin FY, Vural M, Sahin N, Karan A: **Effect of Home-Based Exercise Program on Physical Function and Balance in Older Adults With Sarcopenia: A Multicenter Randomized Controlled Study**. *J Aging Phys Act* 2021, **29**(6):1010-1017.

33. Seo M-W, Jung S-W, Kim S-W, Lee J-M, Jung HC, Song J-K: **Effects of 16 Weeks of Resistance Training on Muscle Quality and Muscle Growth Factors in Older Adult Women with Sarcopenia: A Randomized Controlled Trial**. *International journal of environmental research and public health* 2021, **18**(13).

34. Shahar S, Kamaruddin NS, Badrasawi M, Mohamed Sakian NI, Manaf ZA, Yassin Z, Joseph L: **Effectiveness of exercise and protein supplementation intervention on body composition, functional fitness, and oxidative stress among elderly Malays with sarcopenia**. *Clinical Interventions in Aging* 2013, **8**:1365-1375.

35. Soares Mendes Damasceno G, Teixeira THMM, de Souza VC, Neiva TS, Prudente Pereira K, Teles Landim MdF, de Melo GF, Romao JdFFE, Toledo Nobrega O, de Azevedo Carvalho G: **Acupuncture Treatment in Elderly People with Sarcopenia: Effects on the Strength and Inflammatory Mediators**. *Journal of aging research* 2019, **2019**:8483576.

36. Tamura Y, Kaga H, Abe Y, Yoshii H, Seino H, Hiyoshi T, Kuribayashi N, Inoue I, Watada H: **Efficacy and Safety of 5-Aminolevulinic Acid Combined with Iron on Skeletal Muscle Mass Index and Physical Performance of Patients with Sarcopenia: A Multicenter, Double-Blinded, Randomized-Controlled Trial (ALADDIN Study)**. *Nutrients* 2023, **15**(13).

37. Tsekoura M, Billis E, Tsepis E, Dimitriadis Z, Matzaroglou C, Tyllianakis M, Panagiotopoulos E, Gliatis J: **The Effects of Group and Home-Based Exercise Programs in Elderly with Sarcopenia: A Randomized Controlled Trial**. *J Clin Med* 2018, **7**(12).

38. Vasconcelos KS, Dias JM, Araújo MC, Pinheiro AC, Moreira BS, Dias RC: **Effects of a progressive resistance exercise program with high-speed component on the physical function of older women with sarcopenic obesity: a randomized controlled trial**. *Braz J Phys Ther* 2016, **20**(5):432-440.

39. Vezzoli A, Mrakic-Sposta S, Montorsi M, Porcelli S, Vago P, Cereda F, Longo S, Maggio M, Narici M: **Moderate Intensity Resistive Training Reduces Oxidative Stress and Improves Muscle Mass and Function in Older Individuals**. *Antioxidants (Basel)* 2019, **8**(10).

40. Wang R, Liang Y, Jiang J, Chen M, Li L, Yang H, Tan L, Yang M: **Effectiveness of a Short-Term Mixed Exercise Program for Treating Sarcopenia in Hospitalized Patients Aged 80 Years and Older: A Prospective Clinical Trial**. *J Nutr Health Aging* 2020, **24**(10):1087-1093.

41. Wang Z, Xu X, Gao S, Wu C, Song Q, Shi Z, Su J, Zang J: **Effects of Internet-Based Nutrition and Exercise Interventions on the Prevention and Treatment of Sarcopenia in the Elderly**. *Nutrients* 2022, **14**(12):2458‐2469.

42. Wei M, Meng D, Guo H, He S, Tian Z, Wang Z, Yang G, Wang Z: **Hybrid Exercise Program for Sarcopenia in Older Adults: The Effectiveness of Explainable Artificial Intelligence-Based Clinical Assistance in Assessing Skeletal Muscle Area**. *Int J Environ Res Public Health* 2022, **19**(16).

43. Xiao Y, Song D, Fu N, Zhang L, Zhang Y, Shen R, Wang S, Maitiabula G, Zhou D, Liu S *et al*: **Effects of resistance training on sarcopenia in patients with intestinal failure: A randomized controlled trial**. *Clin Nutr* 2023, **42**(10):1901-1909.

44. Yamada M, Kimura Y, Ishiyama D, Nishio N, Otobe Y, Tanaka T, Ohji S, Koyama S, Sato A, Suzuki M *et al*: **Synergistic effect of bodyweight resistance exercise and protein supplementation on skeletal muscle in sarcopenic or dynapenic older adults**. *Geriatrics & gerontology international* 2019, **19**(5):429‐437.

45. Yin Y-H, Liu JYW, Valimaki M: **Dietary behaviour change intervention for managing sarcopenic obesity among community-dwelling older people: a pilot randomised controlled trial**. *BMC geriatrics* 2023, **23**(1):597.

46. Yuenyongchaiwat K, Akekawatchai C: **Increasing walking steps daily plus resistive exercise can improve muscle strength but not muscle mass in sarcopenia older thai people: A randomized controlled trial**. *European Geriatric Medicine* 2022, **13**:S86.

47. Zhu Y, Peng N, Zhou M, Liu P, Qi X, Wang N, Wang G, Wu Z: **Tai Chi and whole-body vibrating therapy in sarcopenic men in advanced old age: a clinical randomized controlled trial**. *European journal of ageing* 2019, **16**(3):273‐282.
